# Associations between weather extremes and faecal contamination along pathogen transmission pathways in rural Bangladeshi households: a prospective observational study

**DOI:** 10.1016/S2542-5196(24)00306-1

**Published:** 2025-01-21

**Authors:** Caitlin G Niven, Mahfuza Islam, Anna Nguyen, Jessica A Grembi, Andrew Mertens, Amy J Pickering, Laura H Kwong, Mahfuja Alam, Debashis Sen, Sharmin Islam, Mahbubur Rahman, Leanne Unicomb, Alan E Hubbard, Stephen P Luby, John M Colford, Benjamin F Arnold, Jade Benjamin-Chung, Ayse Ercumen

**Affiliations:** a Department of Forestry and Environmental Resources, North Carolina State University, Raleigh, NC, USA; b Division of Environmental Health Sciences, School of Public Health, University of California, Berkeley, CA, USA; c Division of Epidemiology and Biostatistics, School of Public Health, University of California, Berkeley, CA, USA; d Department of Epidemiology & Population Health, Stanford University School of Medicine, Stanford, CA, USA; e Division of Infectious Diseases and Geographic Medicine, Stanford University School of Medicine, Stanford, CA, USA; f Department of Civil and Environmental Engineering, University of California, Berkeley, CA, USA; g Chan Zuckerberg Biohub, San Francisco, CA, USA; h Environmental Health and WASH, Health System and Population Studies Division, icddr,b, Dhaka, Bangladesh; i Francis I. Proctor Foundation, University of California, San Francisco, CA, USA

## Abstract

**Background:**

Weather extremes are predicted to influence pathogen exposure but their effects on specific faecal–oral transmission pathways are not well investigated. We evaluated associations between extreme rain and temperature during different antecedent periods (0–14 days) and *Escherichia coli* along eight faecal–oral pathways in rural Bangladeshi households.

**Methods:**

We used data from the WASH Benefits Bangladesh cluster-randomised controlled trial (NCT01590095). *E coli* was enumerated in hand rinses from children younger than 5 years and their mothers, food, stored drinking water, tubewells, captured flies, ponds, and courtyard soil using IDEXX Quanti-Tray/2000 in nine rounds over 3·5 years and spatiotemporally matched to daily weather data. We used generalised linear models with robust standard errors to estimate *E coli* count ratios (ECRs) associated with extreme rain and temperature, defined as greater than the 90th percentile of daily values during the study period.

**Findings:**

A total of 26 659 samples were collected during the study period. Controlling for temperature, extreme rain on the sampling day was associated with increased *E coli* in food (ECR=3·13 [95% CI 1·63–5·99], p=0·0010), stored drinking water (ECR=1·98 [1·36–2·88], p=0·0004), and ponds (ECR=3·46 [2·34–5·11], p<0·0001), and reduced *E coli* in soil (ECR=0·36 [0·24–0·53], p<0·0001). Extreme rain the day before sampling was associated with reduced *E coli* in tubewells (ECR=0·10 [0·02–0·62], p=0·014). Associations were similar for rainfall 1–7 days before sampling and slightly attenuated for rainfall 14 days before sampling. Controlling for rainfall, extreme temperature on the sampling day was associated with increased *E coli* in stored drinking water (ECR=1·49 [1·05–2·12], p=0·025) and food (ECR=3·01 [1·51–6·01], p=0·0020). Associations with temperature were similar for all antecedent periods and particularly pronounced for food. Neither rainfall nor temperature were consistently associated with *E coli* on hands and flies.

**Interpretation:**

In rural Bangladesh, measures to control enteric infections following weather extremes should focus on water treatment and safe storage to reduce contamination of drinking water and food stored at home and on reducing exposure to surface waters.

**Funding:**

Bill & Melinda Gates Foundation, National Institutes of Health, World Bank.

## Introduction

Increased temperature and rainfall are associated with child diarrhoea but the mechanisms behind these associations are not well investigated.^1^, ^2^ Diarrhoea-causing pathogens are transmitted via the faecal–oral route through environmental compartments (eg, water, soil), food, fomites, and vectors (eg, flies), which can be influenced by weather.^3^ Rainfall can increase pathogen loading into waterbodies, soil, and crops while higher temperatures can support viability, reproduction, and incubation.^4^ Increased faecal contamination in drinking water sources has been observed following higher rainfall and temperature.^5^ Higher temperatures are also associated with increased contamination of food stored at home,^6^ while there is mixed evidence on the association between temperature and contamination on raw produce.^7^ Data on weather effects on other faecal–oral transmission pathways are scarce. A single study found lower *Escherichia coli* counts on children's hands following increased temperature but not rainfall in Kenya.^8^ Understanding how weather fluctuations affect different faecal–oral pathways is critical to identify dominant drivers of enteric pathogen transmission in the face of climate change, predict climate influences on child enteric infections and their sequelae, and design and implement climate-resilient interventions.

**Research in context**

**Evidence before this study**

Higher temperatures and levels of rainfall are associated with increased waterborne and vector-borne disease incidence. Pathogens causing enteric infections are transmitted through multiple environmentally mediated pathways and are responsible for a large disease burden among young children, including diarrhoea, subclinical infections, malnutrition, and growth faltering. However, the specific environmental pathways that facilitate increased transmission of enteric pathogens under fluctuating weather conditions have not been well investigated. Understanding how increased rainfall and temperature affect a comprehensive set of faecal–oral pathways is pivotal for identifying dominant drivers of enteric pathogen transmission in a changing climate and informing climate-resilient interventions to interrupt these pathways to improve child health.

We searched Google Scholar for articles published since 2000 using the following search terms: (“climate change” OR weather OR temperature OR heatwave OR rainfall OR precipitation) AND (pathogen OR enteropathogen OR “*Escherichia coli*” OR *“E coli”* OR “faecal indicator” OR “faecal contamination”) AND (environment OR water OR hands OR food OR soil OR flies). A large body of literature has evaluated the effects of rainfall or temperature on water quality and generally found that higher temperatures and magnitudes of rainfall were associated with higher levels of faecal indicator bacteria, such as *Escherichia coli*, in surface and groundwaters, public and private drinking water sources, and drinking water stored at homes. However, studies on the effects of rainfall and temperature on faecal contamination along non-waterborne faecal–oral transmission pathways are scarce. Contamination of food stored at home has been linked to storage temperature. We found only one study on hand contamination with respect to weather, which found lower *E coli* counts on child hands when daily temperatures were higher but no association with rain. No studies have simultaneously assessed the associations between weather events and a comprehensive set of faecal–oral transmission pathways, which are typically described with the F-diagram and can include drinking water (at the source or stored), surface waters, caregiver and child hands, food, soil, and flies.

**Added value of this study**

We spatiotemporally matched historical meteorological data to over 26 000 *E coli* measurements collected in nine rounds over 3·5 years in a randomised controlled trial in rural villages of central Bangladesh. *E coli* was measured across eight pathogen transmission pathways in the domestic environment. To our knowledge, this study is the first to use a large longitudinal dataset of environmental measurements collected over multiple years to investigate how increased rainfall and temperature are associated with faecal contamination across the full span of pathways described by the F-diagram. Our findings can help identify faecal–oral transmission pathways that are susceptible to extreme weather events. Previous meta-analyses have identified *E coli* measured in drinking water and on child hands and fomites as predictive of child diarrhoea and growth deficits; understanding how weather fluctuations influence these and additional faecal–oral pathways can help predict climate change effects on child health outcomes. Identifying the pathways that experience increased faecal contamination at different times following increased rainfall and temperature can also inform what types of interventions need to be emphasised when to effectively reduce child faecal exposures in the context of climate change.

**Implications of all the available evidence**

In our analysis, extreme rainfall within 2 weeks of sampling was associated with increased *E coli* contamination in stored food, stored drinking water, and ponds, and reduced contamination of tubewell water and courtyard soil. Extreme temperature during the same timeframe was associated with increased contamination of stored food and stored drinking water. These findings illuminate environmental mechanisms behind previously reported increases in diarrhoeal diseases associated with extreme rainfall and temperature. Our findings suggest that, as extreme weather events become more common with climate change, intervention efforts to control exposure to faecal contamination in rural Bangladesh should prioritise reducing contamination of stored food and drinking water, such as emphasising household water treatment, safe storage of water and food, and shorter storage and adequate reheating of stored food in the wake of heavy rain and elevated temperatures. Intervention messaging can also target reducing exposure to contaminated surface waters, such as avoiding ponds for domestic chores, bathing, and swimming following heavy rain, akin to recommendations in the USA to avoid surface water recreation within 3 days of rain.

Bangladesh is the world's seventh most climate-vulnerable country,^9^ experiencing climate-related saltwater intrusion, flooding, water and food insecurity, and shifts in infectious disease patterns.^10^ In Bangladesh, the monsoon season and rainfall events have been associated with increased occurrence of infectious diseases, including pneumonia, diarrhoea, and enteric infections.^11^, ^12^ Higher rainfall and temperatures have also been associated with increased faecal contamination of groundwater in Bangladesh,^13^ while higher *E coli* levels have been observed in soil, ponds, groundwater, stored drinking water, food, children's hands, and flies during the monsoon season.^14^ However, the latter study defined seasons based on month rather than location-specific rainfall data. Climate change might influence both the timing and duration of monsoon seasons, supporting the use of data-driven weather definitions.^15^ Here, we aim to assess associations between increased rainfall and temperature and *E coli* contamination in the domestic environment, using environmental samples and daily weather data from rural Bangladesh.

## Methods

### Study design

We used data from the WASH Benefits Bangladesh cluster-randomised controlled trial (NCT01590095), which measured the effects of water, sanitation, hygiene, and nutrition interventions on child diarrhoea and growth.^16^ The trial enrolled 5551 pregnant women in rural villages in four contiguous districts (Gazipur, Mymensingh, Kishoreganj, and Tangail) in central Bangladesh and followed their birth cohort. The study design has been reported.^16^

### Environmental data

The current analysis pooled data from environmental samples from the control, sanitation, and combined water, sanitation, and hygiene intervention arms of the trial, collected longitudinally in nine rounds over 3·5 years. Sampled pathways included hand rinses from children younger than 5 years and their mothers, prepared food for children younger than 5 years (primarily rice) stored at home for later consumption, stored drinking water, source water (groundwater from tubewells), ponds, courtyard soil, and captured flies. Child hand rinses and stored drinking water were collected during all rounds, mother hand rinses during eight rounds, soil and food during three rounds, and tubewell, pond, and fly samples once. Samples were collected by field staff of the International Centre for Diarrhoeal Disease Research, Bangladesh (icddr,b), trained in sterile technique by study investigators.

Samples were processed at the icddr,b field laboratory with Quanti-Tray/2000 using Colilert-18 media (IDEXX Laboratories, Westbrook, ME, USA) and incubated at 44·5°C.^17^ We enumerated the most probable number (MPN) of *E coli* per 100 mL of water, per two hands, per dry gram of food and soil, and per fly. We imputed non-detects as half the lower detection limit (0·5 MPN) and values above the upper detection limit (2419·6 MPN) as 2420 MPN; detection limits per reporting unit differed by sample type due to different dilution factors and moisture content (appendix p 1). Sample collection and processing details have been reported^14^ (appendix p 1).

### Weather data

Daily rainfall and temperature data were obtained from GloH2O's Multi-Source Weighted-Ensemble Precipitation dataset with a 3-h 1·0° resolution and the National Aeronautics and Space Administration's Famine and Early Warning Systems Network Land Data Assimilation System with a daily 0·01° resolution, using our study area's bands of latitude (23·7–25·0°) and longitude (89·8–90·9°).^18^, ^19^ Missing data were imputed using the nearest location. Daily weather data were matched to *E coli* measurements based on sample collection date and GPS coordinates of study households using nearest neighbour matching, and a Cartesian distance was calculated for each selected match.

We generated weather variables for antecedent periods of 0, 1, 2, 7, and 14 days before sample collection. For each period, we calculated the rolling average rainfall (mm) and temperature (°C). We used daily maximum values to tabulate whether heavy or extreme rainfall, extreme heat, or heatwaves occurred during that window. Heavy and extreme rainfall were defined as the 80th or higher and 90th or higher percentiles, respectively, of daily rainfall values for our study region over the 3·5-year study period.^4^, ^12^ Extreme heat was defined as the 90th or higher percentile of daily temperature values over the study period, and a heatwave as daily maximum temperature above the 95th percentile for 3 consecutive days.^20^ For a sensitivity analysis, we defined elevated heat as the 80th or higher percentile of daily temperature values over the study period. We created categorical variables for rainfall (none, some, heavy, and extreme) and temperature (below-median, above-median, and either extreme or elevated) for each antecedent period and binary variables for heatwaves for the 7-day and 14-day periods.

### Statistical analysis

We used generalised additive models to visualise the relationship between continuous rainfall or temperature values and *E coli* counts. We used generalised linear models with a negative binomial error distribution and robust standard errors to estimate *E coli* count ratios (ECRs) using indicator variables for (1) deciles of rainfall or temperature and (2) the weather categories described above, separately by sample type. Models for rainfall controlled for rolling average temperature during the same period, and vice versa. Models also controlled for intervention status (binary variable for intervention *vs* control) because the interventions significantly improved access to and use of hygienic latrines, handwashing materials, and drinking water treatment. Additionally, models controlled for covariates expected to influence *E coli* levels, measured either at the trial's baseline (mother's age and education, number of children younger than 18 years in household, number of individuals in compound, food security, asset-based wealth index, wall and floor materials, drinking water source, minutes to primary water source, and number of cows, goats, and chickens) or at the time of sample collection (child sex and age). Three additional variables were included for stored drinking water models (duration of storage, whether the storage container was covered, and whether the storage container had a narrow mouth), and one additional variable was included for food models (hours since food was prepared). For stored water and food, we tabulated *E coli* levels by storage conditions to identify any protective associations with safe storage. Analyses were conducted in R (version 4.1.2, RStudio 2022.02.1+461).

### Role of the funding source

The funders of the study had no role in study design, data collection, data analysis, data interpretation, or writing of the report.

## Results

Field staff visited 1840 households in the control, sanitation, and combined water, sanitation, and hygiene arms once between July, 2013, and March, 2014, and 720 households in the control and sanitation arms eight times between June, 2014, and December, 2016, resulting in 652 study dates and 7253 study observations (unique combinations of household GPS coordinates and date). Between July, 2013, and December, 2016, we collected 6350 stored water, 2181 stored food, 5397 mother hand rinse, 7092 child hand rinse, 1669 source water, 2538 soil, 822 pond, and 610 fly samples (appendix p 1). Geometric mean *E coli* counts were 7·8 MPN/100 mL in stored drinking water, 5·0 MPN/g in food, 29·5 MPN per two mother hands, 20·5 MPN per two child hands, 0·9 MPN/100 mL in source water, 125 047 MPN/g in soil, 5397 MPN/100 mL in ponds, and 712 MPN per fly (appendix p 2).

Rainfall data were available for all study observations. The mean distance between study households and rain measurements was 0·4 km (range 8·2 m to 0·76 km). Missing temperature data were imputed for two (0·3%) of 652 study dates. Daily rainfall ranged between 0 mm and 157·1 mm. The 80th percentile corresponded to 16·4 mm, and the 90th percentile to 28·2 mm. Daily temperature ranged between 13·1°C and 34·2°C. The 90th percentile corresponded to 30·2°C and the 95th percentile to 30·9°C. Within 14 days before sampling, 2373 (33%) of 7253 study observations experienced extreme rainfall, and 1984 (27%) experienced extreme temperature. A heatwave occurred within the past 14 days for 910 (13%) study observations (appendix p 3). Highest rainfall and temperatures occurred between March and August (appendix p 4).

**Figure 1:**
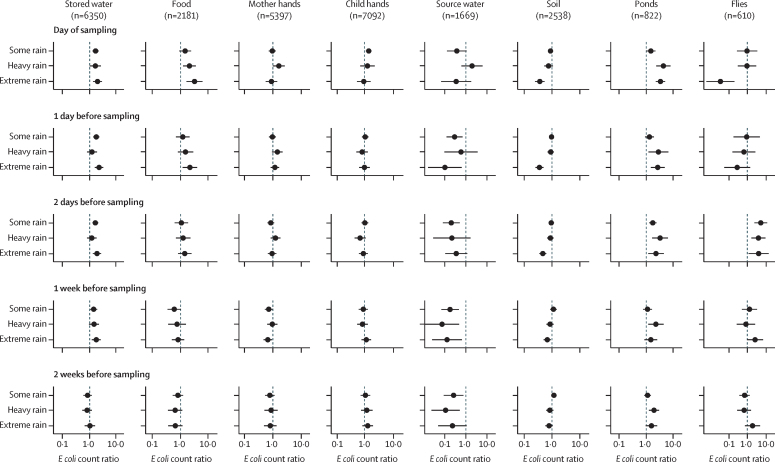
Adjusted *E coli* count ratios by sample type associated with rainfall intensity, compared with no rainfall, during different antecedent periods All adjusted rainfall models controlled for the following variables: rolling mean temperature for the same antecedent period, binary intervention variable (intervention or control), sex of index child, age of index child (in days), number of children younger than 18 years in the household, number of people in the compound, mother's age (in years), mother's educational status, food security category (using the Household Food Insecurity Access Scale), minutes to water source, household having improved walls, household having improved floors, household wealth quintile based on owned assets, number of cows, goats, and chickens or ducks in the compound, and source water origin from a tubewell. Food models included hours since stored food was prepared and stored drinking water models included covered storage container, narrow-mouth storage container, and hours water has been stored. Rainfall intensity was defined as no rain (0 mm), some rain (<16·4 mm), heavy rain (≥16·4 mm and <28·2 mm), and extreme rain (≥28·2 mm). Circles denote point estimates for *E coli* count ratios and error bars indicate the 95% CIs.

Controlling for temperature and confounders, increasing rainfall (analysed both as mm and deciles of rain) on the day of sampling was associated with higher *E coli* counts in food, stored water, and ponds, and lower counts in soil, with statistically significant associations in the higher deciles compared with no rainfall (appendix pp 5–13). Extreme rainfall on the day of sampling was associated with 2-fold to 4-fold higher *E coli* counts in stored water (ECR=1·98 [95% CI 1·36–2·88], p=0·0004), food (ECR=3·13 [1·63–5·99], p=0·0010), and ponds (ECR=3·46 [2·34–5·11], p<0·0001), and lower *E coli* counts in soil (ECR=0·36 [0·24–0·53], p<0·0001) compared with no rainfall (figure 1, appendix p 15). Extreme rainfall the day before sampling was associated with lower *E coli* counts in tubewells (ECR=0·10 [0·02–0·62], p=0·014; figure 1, appendix p 15). Across sample types, associations were similar when rainfall occurred 1–7 days before sampling and attenuated when it occurred 14 days before sampling; for food samples, associations were confined to rainfall occurring 0–1 days before sampling (figure 1, appendix p 15). Rainfall appeared to be associated with higher *E coli* counts in and on flies, but associations were inconsistent overall (figure 1, appendix p 15). There was no consistent association between rainfall and *E coli* on child or mother hands (figure 1, appendix p 15).

**Figure 2:**
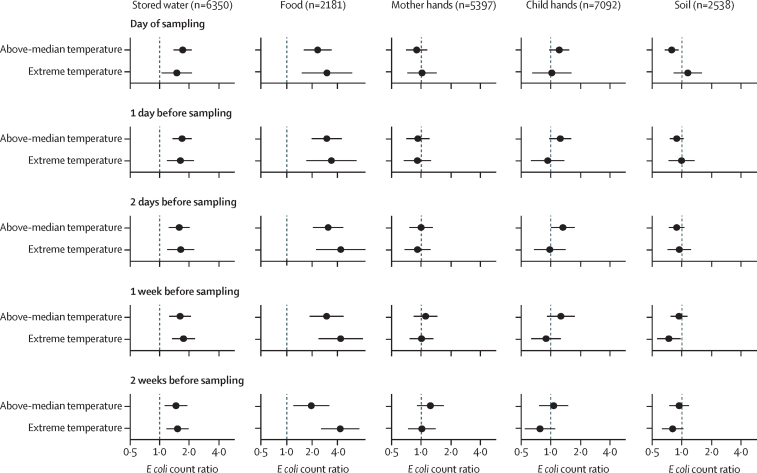
Adjusted *E coli* count ratios by sample type associated with above-median and extreme temperature, compared with below-median temperature, during different antecedent periods All adjusted temperature models controlled for the following variables: rolling mean rainfall for the same antecedent period, binary intervention variable (intervention or control), sex of index child, age of index child (in days), number of children younger than 18 years in the household, number of people in the compound, mother's age (in years), mother's educational status, food security category (using the Household Food Insecurity Access Scale), minutes to water source, household having improved walls, household having improved floors, household wealth quintile based on owned assets, number of cows, goats, and chickens or ducks in the compound, and source water origin from a tubewell. Food models included hours since stored food was prepared and stored drinking water models included covered storage container, narrow-mouth storage container, and hours water has been stored. Temperature categories were defined as below-median temperature (<27·1°C), above-median temperature (≥27·1°C and <30·2°C), and extreme temperature (≥30·2°C). We could not estimate associations between extreme temperature and source water (tubewells), ponds, and flies due to data sparsity. Circles denote point estimates for *E coli* count ratios and error bars indicate the 95% CIs.

Controlling for rainfall and confounders, increasing temperature (analysed both as °C and deciles of temperature) on the day of sampling was associated with higher *E coli* counts in or on food, stored water, child hands, and flies (appendix pp 5–12, 14). For food, stored water, and flies, associations progressively increased in magnitude for each decile increase in temperature, while for child hands, most deciles were associated with a uniform increase in *E coli* counts compared with the lowest decile (appendix p 14). Extreme temperature on the day of sampling was associated with higher *E coli* counts in stored drinking water (ECR=1·49 [95% CI 1·05–2·12], p=0·025) and food (ECR=3·01 [1·51–6·01], p=0·0020) compared with below-median temperature. These associations were similar for all antecedent periods (0–14 days) and particularly pronounced for food (figure 2, appendix p 15). Extreme temperature was not associated with *E coli* on child or mother hands across any antecedent period but above-median temperature 0–2 days before sampling was borderline associated with increased *E coli* on child hands (figure 2, appendix p 15). We could not estimate associations between extreme temperature or heatwaves and contamination of tubewell water, ponds, and flies because only a small number of sampling days for these sample types met these temperature cutoffs. The sensitivity analysis using elevated temperature showed no associations with *E coli* for these sample types, and associations were similar to extreme temperature for stored water and food (appendix p 16). A heatwave within 7 days or 14 days before sampling was associated with lower *E coli* counts in soil (ECR=0·54 [0·38–0·78], p=0·0010 and ECR=0·50 [0·36–0·70], p<0·0001, respectively) but was not associated with *E coli* along other pathways (appendix p 17).

Among 6357 drinking water storage containers, 2882 (45%) had a narrow mouth and 1900 (30%) were covered. The median storage duration was 2 h (IQR 1–4, range 0–600); storage duration was similar on days with and without extreme rain. Mean *E coli* counts were 123 MPN when water was stored for less than 1 h, 148 MPN when stored for between 1 h and 8 h, and 222 MPN when stored for 8 h or longer. Among samples stored for 8 h or longer, the rainfall-associated increase in *E coli* counts was largest when water was stored in an uncovered wide-mouth container and smallest when stored in a covered narrow-mouth container (appendix p 18).

The median food storage time was 3·5 h (IQR 2–5, range 0–29). Mean *E coli* counts were 157 MPN when food was stored for less than 4 h, 346 MPN when stored for between 4 h and 8 h, and 2467 MPN when stored for 8 h or longer. The temperature on food sampling days was between 16·7°C and 33·3°C, within the “danger zone” for bacterial incubation in food (5–60°C) and close to the optimum growth temperature (around 35°C) for enteric bacteria.^21^ Of 1910 food samples collected on days when the temperature was 20°C or higher, 1606 (84%) were stored for 2 h or longer (maximum recommended duration at room temperature^21^); these samples had a mean *E coli* level of 645 MPN, compared with 46 MPN for samples stored for less than 2 h in the same conditions. Of six food samples collected on days when the temperature was 32°C or higher, all six (100%) were stored for 1 h or longer (maximum recommended duration at this temperature^21^); these samples had a mean *E coli* level of 1297 MPN.

## Discussion

Rainfall and temperature significantly influenced *E coli* contamination along multiple faecal–oral pathways in rural Bangladeshi households. Extreme rainfall was associated with increased contamination of stored food, stored drinking water, and ponds, and reduced contamination of tubewell water and courtyard soil. Extreme temperature was associated with increased contamination of stored food and stored drinking water. Associations were more pronounced when these extremes occurred within 7 days of sampling and attenuated when they occurred 14 days before sampling. In a parallel analysis that focused on caregiver-reported diarrhoea and enteropathogen detection in child stool in the WASH Benefits Bangladesh trial, increased rainfall was associated with higher prevalence of diarrhoea and *Cryptosporidium*, enterotoxigenic *E coli* (ETEC), Shiga toxin-producing *E coli* (STEC), *Shigella*, *Campylobacter*, *Aeromonas,* and adenovirus 40/41 detection in stool, and lower prevalence of *Giardia*, sapovirus, and norovirus detection in stool.^12^ Increased temperature was associated with higher prevalence of diarrhoea and STEC, ETEC, and *Cryptosporidium* detection in stool.^12^ Our findings can help interpret the environmental mechanisms behind these associations between weather and child health outcomes.

In our study, samples from the outdoor environment—ponds, groundwater from tubewells, and soil—were sensitive to rainfall but influenced by it in opposite directions (higher contamination of ponds, lower contamination of groundwater and soil). It has been hypothesised that rain releases microorganisms from faecal sources into surface waters via runoff and saturation of the subsurface, and suspends and flushes them out of sediments or soil, while rainfall-associated surface recharge initially delivers contaminants that accumulated on the surface during drier conditions into aquifers but ultimately dilutes them due to increased groundwater volume.^3^, ^22^ Our findings broadly support this conceptual model. In our study setting, faecal sources include leaking or overflowing latrine pits, open defecation (especially by young children), and animal faeces. Reduced soil contamination following heavy rain could also be due to changes in sanitation practices, such as less open defecation or indoor corralling of domestic animals on rainy days. Our findings are consistent with previous evidence of increased faecal contamination of surface waters following rainfall,^23^ but differ from a recent review that found generally higher *E coli* in groundwater with increasing rainfall.^5^ Soils in our study region are primarily alluvial and silt; these soil types pose an effective barrier against pathogen transport.^24^ Groundwater in regions with different hydrogeological features might experience different effects from rainfall. Also, tubewells are an improved water source; groundwater from unimproved or unprotected sources might be more vulnerable to contamination from runoff during heavy rainfall. Among samples collected from the outdoor environment in our study, the only consistently observed association with temperature was reduced *E coli* in soil following heatwaves, consistent with previous evidence of bacterial die-off after prolonged heat exposure.^25^

For samples stored indoors—stored drinking water and prepared food—both extreme rainfall and temperature were associated with increased contamination. Given that rainfall did not adversely affect groundwater quality at the source, increased contamination of stored water likely stemmed from rainfall-associated bacterial intrusion or growth in storage containers during collection, transport, or storage. Mechanisms for this might include splashing of faecally contaminated soil or mud into storage containers while filling them at the tubewell in the rain or during storage, less thorough or frequent cleaning of storage containers to avoid going outside to the tubewell, or use of runoff-contaminated pond water to wash storage containers and utensils that will contact stored water; similar mechanisms could also explain the increased contamination of stored food following increased rainfall. In our study, drinking water was stored at home longer (maximum 600 h) than food (maximum 29 h); this might explain why associations with food contamination were confined to rainfall occurring 0–1 days before sampling, whereas associations with stored water contamination persisted for rainfall occurring up to a week before. The increase in stored water contamination associated with rainfall appeared largest when water was stored unsafely (in uncovered wide-mouth container) and smallest when stored safely (in covered narrow-mouth container). A previous study in Bangladesh found that safe storage of tubewell water in a container with a narrow mouth, tight-fitting lid, and spigot significantly improved stored water quality and reduced diarrhoea.^26^ In a recent study in Kenya, rainfall-associated increases in *E coli* in stored drinking water were mitigated if households treated their water.^8^ Our findings suggest that water treatment and safe storage of drinking water and food are especially important immediately following extreme rainfall. Household-level water treatment interventions often have low uptake; focusing treatment recommendations on these high-risk times might help achieve higher uptake and maximise health benefits.

Higher *E coli* counts in stored water and food associated with increased ambient temperatures may be due to increased bacterial growth during storage under warm conditions. Higher food storage temperature has been associated with increased contamination, with substantial increases when foods are stored for longer than 4 h after preparation.^6^ WHO recommends that food not be stored for more than 2 h at room temperature, and not be left out for more than 1 h when ambient temperatures exceed 32°C.^21^ In our study, *E coli* levels in food stored for 1 h or longer doubled at ambient temperatures of 32°C or higher versus 20°C. Additional mechanisms for temperature-associated contamination of stored drinking water and food could include less frequent reheating of food to avoid additional heat exposure or less frequent or thorough cleaning of storage containers and utensils to avoid stepping outdoors in the heat. Our findings emphasise the importance of minimising the duration of food storage during extreme ambient temperatures to reduce pathogen growth, as well as adequate reheating before consumption.

We did not find a consistent association between rainfall or temperature and contamination of child or mother hands. In Kenya, children had less *E coli* on their hands on days with higher temperatures, while there was no association with rainfall.^8^ Others have hypothesised that children might have cleaner hands during higher rainfall and temperatures because they might play outside less or have more water for handwashing.^8^ Children in our study were young (85% ≤3 years, 30% ≤1 year). Children in this age range have frequent hand contact with objects, indoor surfaces, and floors; unmeasured contamination from these pathways may have obscured associations with weather conditions. In Bangladesh, *E coli* on child hands was associated with subsequent child diarrhoea,^27^ and hand mouthing was a dominant contributor to *E coli* ingestion by children younger than 3 years.^28^ Lack of associations between weather and child hand contamination in our analysis indicates that increases in child diarrhoea following increased rainfall or temperature might not be mediated by faecal contamination of child hands. We are unaware of previous studies on associations between weather and caregiver hand contamination. *E coli* levels on caregiver hands are highly temporally variable;^29^ our findings indicate that the contribution of weather to contamination of mother hands might be negligible compared with other dominant factors (eg, domestic chores or contact with domestic animals). Additionally, although caregiver hand contamination is predictive of stored water contamination,^30^ our findings indicate that the higher *E coli* levels we observed in stored drinking water and food after increased rainfall or temperature are not due increased mother or child hand contamination.

This study spatiotemporally matched daily weather data to more than 26 000 *E coli* measurements. Study strengths include a large sample size to allow statistical precision, comprehensive set of sample types to evaluate associations with weather across the F-diagram, longitudinal measurements over multiple years to capture a range of weather conditions, and use of daily weather data as opposed to regional definitions of seasonality.

Our study also had limitations. The nearest available rainfall data were located up to 0·76 km from households, and temperature data were aggregated over 0·01 × 0·01° pixels, potentially not reflecting exact conditions at sampling sites. Any resulting weather misclassification would be non-differential with respect to our outcomes and bias findings towards the null. *E coli* as a faecal indicator is imperfectly correlated with pathogen presence and can originate from non-faecal sources.^25^ Therefore, our analysis might not capture the weather responses of pathogens, especially non-bacterial pathogens. For example, higher temperatures are associated with reduced viral infections and increased protozoan infections.^3^ Future research should investigate weather effects on specific pathogens in the environment. However, phenotypic and genotypic characterisation of a subset of hand rinse and soil samples from our study indicated that *E coli* isolated from these sample types were indistinguishable from *E coli* isolated from cow, chicken, and human faeces collected in the study area, indicating that the *E coli* in the environmental samples were of faecal origin.^31^ Furthermore, 22% of mother hands, 32% of child hands, 37% of stored drinking water, and 60% of soil samples harboured at least one *E coli* virulence gene, indicating disease-causing potential through these pathways.^32^ *E coli* on child hands has been associated with subsequent child diarrhoea in Bangladesh,^27^ and a meta-analysis found that *E coli* contamination of drinking water, child hands, and fomites was associated with increased diarrhoea and reduced linear growth.^33^ Therefore, our findings on the associations between weather and *E coli* in different sample types in the domestic environment are likely informative of child health risks associated with these pathways under extreme weather conditions.

Bangladesh experiences a high burden of enteric infections; therefore, understanding how weather extremes influence infectious disease transmission in this setting can inform other countries with similar infection burdens. However, several factors might limit the generalisability of our study. Bangladesh has a distinct monsoon season that delivers 80% of the annual rainfall; the associations between weather and environmental contamination could be different in settings with different meteorology and hydrogeology. Our study was conducted in a rural environment with high on-premise access to improved water sources and pit latrines but commonly practised child open defecation and unsafe management of child and animal faeces. Associations between weather and faecal–oral transmission pathways might vary in urban or densely populated areas and settings with varying water and sanitation infrastructure and waste management practices. Additionally, it is possible that the relationship between weather and *E coli* is spatially variable within our study area, but we did not explore this heterogeneity as the WASH Benefits Bangladesh trial was conducted in contiguous areas with relatively homogeneous environmental conditions.

In conclusion, our findings provide insight on the environmental mechanisms behind a body of literature indicating increased diarrhoeal illness during warmer or rainy periods. As extreme weather events become more frequent and severe, it is important to identify and target the transmission pathways most impacted by these events. Our findings indicate that drinking water and food stored in the home and surface waters are vulnerable to contamination from increased rainfall or temperature. Preventive messaging should consider targeting the immediate week, especially the first 2 days, after extreme rainfall and temperatures to emphasise protective practices (eg, water treatment, safe storage of drinking water and prepared food, adequate reheating of stored food) to reduce exposure to faecal organisms. Following extreme rainfall, avoiding bathing or swimming in surface waterbodies to prevent accidental ingestion and not using surface water for cooking and washing utensils that will contact food or stored water can also help reduce exposures.

### Contributors

### Data sharing

De-identified individual participant data collected for the study, a data dictionary defining each field in the set, and data analysis scripts are available upon publication at Open Science Framework: https://osf.io/6u7cn/. The data are publicly available with no restriction.

## Declaration of interests

We declare no competing interests.
