# Supplementary material for: Associations between weather extremes and faecal contamination along pathogen transmission pathways in rural Bangladeshi households: a prospective observational study
Source: Lancet Planet Health. 2025 Jan 21;9(1):e5–e13. doi: 10.1016/S2542-5196(24)00306-1 (PMC11755722; doi:10.1016/S2542-5196(24)00306-1)
Supplement: Supplementary appendix [file mmc1.pdf]

THE LANCET
Planetary Health

Supplementary appendix

This appendix formed part of the original submission and has been peer reviewed. We post it as supplied by the authors.

Supplement to: Niven CG, Islam M, Nguyen A, et al. Associations between weather extremes and faecal contamination along pathogen transmission pathways in rural Bangladeshi households: a prospective observational study. *Lancet Planet Health* 2025; **9:** e5–13.

Appendix

Associations between weather extremes and faecal contamination along pathogen transmission pathways in rural Bangladeshi households: A prospective observational study

Caitlin G. Niven[1*], BA; Mahfuza Islam[2], MPH; Anna Nguyen[3], MPH; Jessica A. Grembi[3], PhD; Andrew Mertens[4], PhD; Amy J. Pickering[5,6], PhD; Laura H. Kwong[2], PhD; Mahfuja Alam[7], MS; Debashis Sen[7], MS; Sharmin Islam[7], MS; Mahbubur Rahman[7], MBBS; Leanne Unicomb[7], PhD; Alan E. Hubbard[4]†, PhD; Stephen P. Luby[8]†, MD; John M. Colford, Jr.[4]†, PhD; Benjamin F. Arnold[9], PhD; Jade Benjamin-Chung[3,6], PhD; Ayse Ercumen[1], PhD

1. Department of Forestry and Environmental Resources, North Carolina State University, Raleigh, NC 27695
2. Division of Environmental Health Sciences, School of Public Health, University of California, Berkeley, CA 94720
3. Department of Epidemiology & Population Health, Stanford University School of Medicine, Stanford, CA 94305
4. Division of Epidemiology and Biostatistics, School of Public Health, University of California, Berkeley, CA 94720
5. Department of Civil and Environmental Engineering, University of California, Berkeley, CA 94720
6. Chan Zuckerberg Biohub, San Francisco, CA 94158
7. Environmental Health and WASH, Health System and Population Studies Division, icddr,b Dhaka -1212, Bangladesh
8. Division of Infectious Diseases and Geographic Medicine, Stanford University School of Medicine, Stanford, CA 94305
9. Francis I. Proctor Foundation, University of California, San Francisco, CA 94143

*Corresponding Author: Caitlin G. Niven, Jordan Hall Addition 2225, Raleigh, NC, 27606,
+1 (415) 425 2910, cgniven@ncsu.edu

† Indicates full professor

Funding Sources: Bill & Melinda Gates Foundation, National Institutes of Health (NIH), World Bank

Table of Contents

Table S1: *E. coli* reporting units and detection limits by sample type 1

Table S2: Number of samples collected by sample type and study round 1

Text S1: Sample collection and processing details 1

Table S3: Number of study observations and geometric mean *E. coli* counts by sample type for each weather category across different antecedent timeframes 2

Table S4: Percentages of study observations within each weather category across different antecedent timeframes 3

Figure S1: Daily precipitation and temperature, and monthly mean most probable number (MPN) of *E. coli* by sample type across the study period 4

Figure S2: Adjusted generalized additive model plot of *E. coli* counts on child hands vs. continuous rainfall and temperature on the day of sample collection 5

Figure S3: Adjusted generalized additive model plot of *E. coli* counts on mother hands vs. continuous rainfall and temperature on the day of sample collection. 6

Figure S4: Adjusted generalized additive model plot of *E. coli* counts in stored water vs. continuous rainfall and temperature on the day of sample collection 7

Figure S5: Adjusted generalized additive model plot of *E. coli* counts in soil vs. continuous rainfall and temperature on the day of sample collection 8

Figure S6: Adjusted generalized additive model plot of *E. coli* counts in food vs. continuous rainfall and temperature on the day of sample collection 9

Figure S7: Adjusted generalized additive model plot of *E. coli* counts in ponds vs. continuous rainfall and temperature on the day of sample collection 10

Figure S8: Adjusted generalized additive model plot of *E. coli* counts in/on flies vs. continuous rainfall and temperature on the day of sample collection 11

Figure S9: Adjusted generalized additive model plot of *E. coli* counts in tubewell water vs. continuous rainfall and temperature on the day of sample collection. 12

Figure S10: Adjusted *E. coli* count ratios associated with deciles of rain, compared to no rain, on the day of sampling 13

Figure S11: Adjusted *E. coli* count ratios associated with deciles of temperature, compared to 1st decile of temperature, on the day of sampling 14

Table S5: Adjusted *E. coli* count ratios by sample type associated with each weather category across different antecedent timeframes 15

Figure S12: Adjusted *E. coli* count ratios by sample type associated with above-median and elevated temperature, compared to below-median temperature, during different antecedent periods 16

Figure S13: Adjusted *E. coli* count ratios by sample type associated with heatwaves within 7 and 14 days 17

Table S6: Mean *E. coli* (MPN/100 mL) in stored drinking water by storage container type and rainfall, among samples stored for at least 8 hours 18

Table S1. ***E. coli*** **reporting units and detection limits by sample type**

	Unit	Lower Limit (MPN)	Upper Limit (MPN)
Stored water	100 mL	1	2419.6
Food	1 dry gram	1–2 [a]	2,494 – 48,392 [a]
Mother hands	2 hands	5	12,098
Child hands	2 hands	5	12,098
Source water	100 mL	1	2,419.6
Soil	1 dry gram	1,000–1887 [b]	2.42 x 10^6– 4.56 x 10^6 [b]
Ponds	100 mL	100	241,960
Flies	1 fly	100	241,960

[a] Given a food moisture content range of 3-95%, a lower limit of 1 MPN and upper limit of 2,419·6 MPN per wet gram
[b] Given a soil moisture content range of 0-47%, a lower limit of 1,000 MPN and upper limit of 2,419,600 per wet gram

Table S2. Number of samples collected by sample type and study round

Round	Round 1	Round 2	Round 3	Round 4	Round 5	Round 6	Round 7	Round 8	Round 9	Total
Stored water	1623	641	592	571	599	581	582	574	587	6350
Food	1646	0	0	329	206	0	0	0	0	2181
Mother hands	0	720	705	684	682	668	662	643	633	5397
Child hands	1768	720	705	682	673	653	650	626	615	7092
Source water	1669	0	0	0	0	0	0	0	0	1669
Soil	1795	0	0	402	341	0	0	0	0	2538
Ponds	822	0	0	0	0	0	0	0	0	822
Flies	610	0	0	0	0	0	0	0	0	610
Total	9933	2081	2002	2668	2501	1902	1894	1843	1835	26659

Text S1. Sample collection and processing details

Stored drinking water was collected by asking the participant to provide a glass of water from their storage container, meant for children under five, and approximately 150 mL was poured into a sterile Whirlpak bag. Hand rinse samples were taken from index children - those born to women pregnant at enrollment - or the youngest available child under five, and their mothers. Each hand was placed into a sterile Whirlpak bag prefilled with 250 mL of distilled water, massaged from the outside for 15 seconds, shaken for 15 seconds, and then repeated for the other hand. Soil samples were collected from a 30 cm x 30 cm area in the courtyard near the household entrance. A sterile plastic scoop was used to scrape around 50 g from the top layer of soil into a sterile Whirlpak bag. Stored food samples were collected by asking participants to provide a small amount of food as they would for children under five, which was scooped into a 50 mL sterile tube using a sterile spoon. Tubewell water was collected by removing attached materials from the mouth, flushing the tubewell via pumping five times, and collecting 250 mL. Pond water was collected by submerging a Whirlpak bag into the pond at the household's usual access point to collect 250 mL. Sticky fly tape strips were hung in the food preparation area for 3-6 hours for fly collection. One fly was removed using sterile tweezers and placed into a Whirlpak bag. Clean gloves were worn during all sample collections, and quality control procedures included 10% field blanks and duplicates. Samples were placed on ice and transported to the field laboratory at the International Centre for Diarrhoeal Disease Research, Bangladesh (icddr,b), for processing within 24 hours of collection using IDEXX Quanti-Tray/2000. Stored drinking water was analyzed in 100 mL undiluted aliquots and hand rinses were diluted to 50 mL with 50 mL of distilled water. 20 grams of soil was homogenized with 200 mL distilled water and then diluted to create a 100 mL aliquot. Another 5 g of soil was reserved to determine moisture content by oven-drying at 110 °C for 24 hours. Stored food samples were homogenized by mixing 10 g of food with 100 mL of distilled water and then diluted 1:10. Moisture content for food was carried out in the same manner as for soil. Fly samples were homogenized by mixing with 100 mL of distilled water and diluted 1:100. *E. coli* were enumerated using the most probable number (MPN) method after incubating for 18 hours with Colilert-18 media at 44.5 °C.

Table S3. Number of study observations and geometric mean *E. coli* counts by sample type for each weather category[a] across different antecedent timeframes

	Stored Water (N=6350)		Food (N=2181)		Mother Hands (N = 5397)		Child Hands (N= 7092)		Source Water (N=1669)		Soil (N=2538)		Ponds (N=822)		Flies (N=610)	
	% (n)	Geometric Mean (SD)	% (n)	Geometric Mean (SD)	% (n)	Geometric Mean (SD)	% (n)	Geometric Mean (SD)	% (n)	Geometric Mean (SD)	% (n)	Geometric Mean (SD)	% (n)	Geometric Mean (SD)	% (n)	Geometric Mean (SD)
**Day of**																
No Rain	52.8% (3356)	5.31 (9.95)	49.4% (1078)	1.63 (14.94)	49.2% (2656)	31.93 (9.88)	52.3% (3706)	18.66 (9.24)	62.1% (1037)	0.82 (3.90)	48% (1218)	121285.80 (13.56)	75.9% (624)	4994.44 (5.84)	56.2% (343)	432.50 (19.49)
Some Rain	37.8% (2401)	10.81 (12.10)	40.4% (881)	11.81 (27.62)	39.8% (2146)	25.17 (9.75)	38.4% (2722)	22.41 (10.34)	33.6% (560)	1.10 (4.86)	41.8% (1062)	139114.00 (10.22)	19.1% (157)	4837.13 (6.78)	39.2% (239)	1429.36 (23.52)
Heavy Rain	4.3% (270)	14.77 (12.61)	5.2% (114)	41.18 (23.45)	4.9% (267)	35.71 (12.90)	4.4% (313)	24.14 (9.87)	2.8% (47)	1.54 (9.10)	5.2% (133)	111340.10 (9.58)	3.4% (28)	27879.14 (4.44)	2.6% (16)	1544.76 (40.20)
Extreme Rain	5.1% (323)	21.60 (11.20)	5% (108)	30.68 (26.77)	6.1% (328)	36.99 (9.26)	4.9% (351)	24.72 (8.91)	1.5% (25)	1.52 (5.24)	4.9% (125)	77025.78 (7.58)	1.6% (13)	24471.59 (6.32)	2% (12)	369.71 (10.10)
Below Median Temp	52.7% (3345)	5.70 (10.29)	62.5% (1363)	2.09 (17.52)	43.7% (2360)	32.88 (9.80)	52.5% (3721)	17.68 (9.19)	79.1% (1320)	0.90 (4.29)	61.5% (1562)	138924.20 (11.58)	90.5% (744)	5550.86 (6.09)	74.4% (454)	579.08 (21.60)
Above Median Temp	38.4% (2442)	11.30 (12.25)	32.2% (703)	19.48 (28.02)	44.2% (2384)	27.23 (9.94)	38.3% (2719)	23.39 (10.23)	20.7% (346)	1.06 (4.79)	33% (838)	97628.63 (10.68)	9.1% (75)	4420.02 (7.12)	25.1% (153)	1337.50 (22.90)
Extreme Temp	8.9% (563)	9.96 (10.96)	5.3% (115)	32.96 (19.41)	12.1% (653)	26.47 (10.51)	9.2% (652)	28.07 (9.76)	0.2% (3)	0.50 (1.00)	5.4% (138)	170818.30 (16.90)	0.4% (3)	761.89 (1.66)	0.5% (3)	304.56 (22.86)
**1 Day**																
No Rain	45.5% (2888)	4.88 (9.59)	44.8% (978)	1.36 (13.63)	40.9% (2206)	32.58 (9.72)	45.1% (3196)	18.30 (9.33)	58% (968)	0.82 (3.90)	42.9% (1089)	112160.30 (14.03)	70.5% (580)	4893.64 (6.06)	51% (311)	408.03 (18.17)
Some Rain	39.6% (2514)	10.22 (12.26)	39.5% (861)	10.21 (26.57)	41.7% (2252)	24.99 (9.69)	39.8% (2824)	22.32 (10.09)	33.9% (566)	1.07 (4.90)	40.7% (1033)	159589.20 (10.04)	21.8% (179)	5288.68 (5.82)	42.3% (258)	1419.65 (25.28)
Heavy Rain	7% (446)	11.47 (10.91)	8.2% (179)	28.51 (23.57)	8% (432)	28.96 (11.54)	7.2% (512)	21.62 (9.81)	4.9% (81)	1.29 (7.07)	8.2% (207)	121600.30 (11.03)	5.4% (44)	13847.16 (6.23)	3.8% (23)	802.28 (30.00)
Extreme Rain	7.9% (502)	21.15 (11.36)	7.5% (163)	38.26 (25.58)	9.4% (507)	40.22 (10.46)	7.9% (560)	24.83 (9.35)	3.2% (54)	1.17 (3.82)	8.2% (209)	67865.91 (7.23)	2.3% (19)	14685.42 (9.01)	2.9% (18)	468.43 (12.00)
Below Median Temp	48.3% (3065)	5.42 (10.12)	57.2% (1247)	1.82 (14.43)	39.2% (2118)	32.35 (9.58)	47.9% (3396)	17.51 (9.12)	74.5% (1243)	0.90 (4.27)	60% (1421)	137132.80 (12.01)	88.2% (725)	5526.74 (6.10)	70.7% (431)	551.34 (20.99)
Above Median Temp	39.9% (2537)	11.33 (12.13)	35.7% (778)	17.73 (30.44)	45% (2426)	28.61 (10.22)	40.1% (2844)	23.04 (10.32)	25.2% (420)	1.04 (4.80)	36.6% (930)	107046.10 (10.09)	11.3% (93)	4989.69 (6.64)	28.4% (173)	1385.66 (23.97)
Extreme Temp	11.8% (748)	9.71 (11.40)	7.1% (156)	26.66 (19.32)	15.8% (853)	25.56 (10.15)	12% (852)	26.35 (9.49)	0.3% (6)	0.50 (1.00)	7.4% (187)	134374.80 (16.45)	0.5% (4)	458.58 (2.99)	0.9% (6)	316.42 (17.45)
**2 Days**																
No Rain	41.8% (2655)	4.79 (9.51)	43.5% (950)	1.30 (13.33)	36.5% (1971)	33.18 (9.72)	41.5% (2945)	17.58 (9.21)	56.9% (949)	0.82 (3.90)	41.5% (1054)	112622.60 (14.14)	68.2% (561)	4715.59 (6.06)	50% (305)	389.66 (17.36)
Some Rain	38.3% (2430)	9.73 (12.24)	33.7% (735)	9.83 (26.93)	41.4% (2234)	24.82 (9.72)	38.2% (2712)	23.95 (10.25)	28.3% (473)	1.09 (4.94)	35.1% (892)	152067.20 (10.67)	18.9% (155)	5134.47 (5.84)	35.2% (215)	1325.16 (23.41)
Heavy Rain	8.9% (563)	11.47 (11.28)	10.7% (233)	23.75 (22.31)	9.3% (502)	30.18 (11.73)	8.9% (630)	18.94 (9.39)	7.7% (128)	1.02 (5.28)	10.6% (268)	150455.00 (10.22)	8.5% (70)	11793.15 (6.45)	7.2% (44)	898.60 (34.03)
Extreme Rain	11.1% (702)	16.70 (11.52)	12.1% (263)	23.29 (27.78)	12.8% (690)	36.04 (9.89)	11.4% (805)	22.98 (9.45)	7.1% (119)	1.20 (5.03)	12.8% (324)	88017.53 (7.50)	4.4% (36)	12008.60 (5.99)	7.5% (46)	1704.53 (27.98)
Below Median Temp	46.3% (2940)	5.32 (10.11)	54.6% (1192)	1.71 (14.84)	37.6% (2027)	32.31 (9.46)	45.9% (3258)	17.26 (9.10)	71.7% (1197)	0.85 (4.01)	53.4% (1355)	134851.00 (12.33)	87.1% (716)	5586.98 (6.10)	67.5% (412)	529.80 (20.71)
Above Median Temp	39.9% (2533)	11.18 (12.02)	36.2% (789)	16.67 (30.30)	44.1% (2382)	29.02 (10.36)	40% (2839)	23.35 (10.33)	27.4% (457)	1.18 (5.42)	37.3% (947)	112930.10 (9.66)	12.3% (101)	4643.53 (6.57)	30.3% (185)	1311.68 (23.92)
Extreme Temp	13.8% (877)	9.93 (11.50)	9.2% (200)	23.94 (21.45)	18.3% (988)	25.34 (10.00)	14% (995)	25.13 (9.47)	0.9% (15)	0.64 (2.55)	9.3% (236)	122036.30 (16.00)	0.6% (5)	803.87 (4.82)	2.1% (13)	1406.45 (26.72)
**7 Days**																
No Rain	31.3% (1989)	4.31 (9.35)	38.8% (847)	1.23 (13.08)	24.7% (1332)	33.58 (9.94)	31.3% (2217)	15.17 (8.93)	51.7% (862)	0.83 (4.02)	36.6% (929)	109019.50 (14.39)	61.6% (506)	4740.81 (6.21)	44% (268)	386.05 (17.94)
Some Rain	34.9% (2216)	8.33 (11.11)	20.3% (442)	6.73 (24.62)	41% (2212)	27.46 (9.76)	34.8% (2467)	25.35 (9.63)	16% (267)	0.93 (4.58)	23.2% (589)	149509.50 (13.03)	13.6% (112)	4887.53 (5.28)	19.3% (118)	729.95 (19.88)
Heavy Rain	12% (763)	13.28 (12.47)	13.8% (300)	20.18 (22.61)	12.1% (655)	29.19 (11.24)	11.9% (842)	19.55 (10.06)	11% (184)	0.95 (4.37)	13.7% (348)	130922.60 (11.03)	12.3% (101)	9288.00 (6.31)	10% (61)	897.81 (21.09)
Extreme Rain	21.8% (1382)	12.27 (12.12)	27.1% (592)	14.35 (29.24)	22.2% (1198)	29.21 (9.67)	22.1% (1566)	23.23 (10.25)	21.3% (356)	1.20 (5.09)	26.5% (672)	126208.70 (7.50)	12.5% (103)	6677.73 (6.38)	26.7% (163)	1755.19 (28.13)
Below Median Temp	42.4% (2691)	4.90 (9.77)	51.4% (1120)	1.58 (14.39)	33.6% (1813)	32.35 (9.31)	41.9% (2972)	16.66 (8.94)	67.5% (1126)	0.83 (3.93)	49.9% (1267)	131726.60 (12.86)	82.2% (676)	5538.48 (6.03)	60.3% (368)	467.07 (19.83)
Above Median Temp	37.8% (2400)	10.80 (12.04)	34.8% (759)	13.03 (29.78)	40% (2158)	30.12 (10.37)	37.9% (2691)	23.51 (10.57)	31.6% (528)	1.19 (5.36)	35.8% (908)	128507.50 (9.14)	17.2% (141)	5102.58 (6.80)	37.5% (229)	1349.39 (23.96)
Extreme Temp	19.8% (1259)	11.28 (11.67)	13.8% (302)	30.69 (21.65)	26.4% (1426)	25.33 (10.15)	20.1% (1429)	24.58 (9.33)	0.9% (15)	0.64 (2.55)	14.3% (363)	97396.32 (13.89)	0.6% (5)	803.87 (4.82)	2.1% (13)	1406.45 (26.72)
No Heatwave	90.6% (5751)	7.63 (11.29)	93% (2028)	4.43 (25.44)	86.9% (4689)	30.99 (10.03)	90.1% (6392)	20.42 (9.76)	100% (1669)	0.93 (4.40)	92.5% (2347)	134597.40 (11.23)	100% (822)	5397.41 (6.18)	100% (610)	712.12 (22.30)
Heatwave	9.4% (599)	9.64 (11.64)	7% (153)	22.37 (14.93)	13.1% (708)	21.11 (9.25)	9.9% (700)	21.65 (9.01)	0% (0)	[b]	7.5% (191)	50619.76 (14.25)	0% (0)	[b]	0% (0)	[b]
**14 Days**																
No Rain	18.8% (1195)	4.19 (9.63)	23.4% (511)	1.05 (12.29)	14.5% (783)	35.88 (9.71)	18.8% (1334)	14.70 (8.75)	32.5% (543)	0.89 (4.59)	22.7% (576)	89903.28 (15.62)	37% (304)	4239.19 (6.50)	25.7% (157)	410.14 (19.49)
Some Rain	34.1% (2164)	6.35 (10.19)	19.1% (416)	2.06 (17.50)	38.5% (2076)	28.32 (9.78)	33.8% (2396)	23.42 (9.31)	20.5% (342)	0.73 (2.97)	20.7% (525)	142281.80 (13.69)	24.3% (200)	4693.61 (6.00)	20.7% (126)	359.45 (16.39)
Heavy Rain	14.7% (934)	10.00 (11.60)	19.8% (432)	9.28 (22.45)	13.1% (706)	26.88 (10.55)	14.5% (1027)	18.53 (10.72)	18.5% (308)	0.89 (3.98)	19.3% (491)	117548.00 (12.02)	19.7% (162)	8391.78 (5.69)	17.7% (108)	672.08 (18.97)
Extreme Rain	32.4% (2057)	12.40 (12.34)	37.7% (822)	14.67 (28.63)	33.9% (1832)	29.37 (10.03)	32.9% (2335)	22.73 (9.99)	28.5% (476)	1.21 (5.41)	37.3% (946)	146942.20 (8.18)	19% (156)	6536.77 (5.81)	35.9% (219)	1612.75 (26.41)
Below Median Temp	38.7% (2458)	4.67 (9.72)	49.7% (1083)	1.51 (14.10)	29.5% (1590)	32.37 (9.18)	38.3% (2714)	15.99 (8.94)	65.5% (1093)	0.82 (3.94)	47.5% (1205)	125198.60 (13.20)	78.8% (648)	5333.28 (6.03)	58.7% (358)	477.95 (19.88)
Above Median Temp	34.3% (2176)	10.63 (11.81)	28.6% (624)	9.38 (27.31)	36.4% (1966)	32.89 (10.60)	34.3% (2433)	23.40 (10.44)	27.5% (459)	1.25 (5.33)	30.6% (776)	137487.90 (9.19)	16.8% (138)	5539.64 (6.51)	33.6% (205)	1043.56 (22.58)
Extreme Temp	27% (1716)	10.96 (11.74)	21.7% (474)	32.72 (24.55)	34.1% (1841)	24.17 (9.87)	27.4% (1945)	23.47 (9.54)	7.0% (117)	0.89 (4.55)	21.9% (557)	109283.10 (11.79)	4.4% (36)	6057.94 (8.14)	7.7% (47)	2803.68 (29.01)
No Heatwave	87.8% (5576)	7.57 (11.27)	91.6% (1997)	4.29 (25.22)	83.2% (4490)	31.41 (10.04)	87.4% (6195)	20.34 (9.75)	100% (1669)	0.93 (4.40)	90.9% (2308)	135931.00 (11.21)	100% (822)	5397.41 (6.18)	100% (610)	712.12 (22.30)
Heatwave	12.2% (774)	9.64 (11.71)	8.4% (184)	24.24 (16.06)	16.8% (907)	21.49 (9.37)	12.6% (897)	21.91 (9.27)	0% (0)	[b]	9.1% (230)	54121.62 (13.77)	0% (0)	[b]	0% (0)	[b]

[a] Weather classifications are as follows: no rain (0 mm), some rain (<16·4 mm), heavy rain (≥16·4 and <28·2 mm), extreme rain (≥28·2 mm), below-median temperature (<27·1˚C), above-median temperature (≥27·1 and <30·2˚C), extreme temperature (≥ 30·2˚C), no heatwave (<30·9˚C), heatwave (≥ 30·9˚C for 3 consecutive days).
[b] We could not estimate effects of heatwaves on source water (tubewells), ponds and flies due to data sparsity.

Table S4. **Percentages of study observations within each weather category[a] across different antecedent timeframes. Study observation refers to unique combinations of household GPS coordinates and sample collection dates (total n=7253)**

	**0 Days**	**1 Day**	**2 Days**	**7 Days**	**14 Days**
No Rain	52.4% (3803)	45.1% (3274)	41.6% (3014)	31.3% (2270)	18.8% (1361)
Some Rain	38.2% (2773)	39.8% (2888)	38.3% (2776)	34.8% (2525)	34% (2468)
Heavy Rain	4.4% (319)	7.2% (521)	8.8% (641)	11.8% (858)	14.5% (1051)
Extreme Rain	4.9% (358)	7.9% (570)	11.3% (822)	22.1% (1600)	32.7% (2373)
Below Median Temp	52.6% (3812)	48.0% (3483)	46.1% (3344)	42.1% (3051)	38.4% (2785)
Above Median Temp	38.3% (2778)	40.0% (2903)	39.9% (2896)	37.9% (2748)	34.3% (2486)
Extreme Temp	9.1% (663)	12.0% (867)	14.0% (1013)	20.0% (1454)	27.3% (1982)
No Heatwave	--	--	--	90.2% (6543)	87.5% (6343)
Heatwave	--	--	--	9.8% (710)	12.5% (910)

[a] Weather classifications are as follows: no rain (0 mm), some rain (<16·4 mm), heavy rain (≥16·4 and <28·2 mm), extreme rain (≥28·2 mm), below-median temperature (<27·1˚C), above-median temperature (≥27·1 and <30·2˚C), extreme temperature (≥ 30·2˚C), no heatwave (<30·9˚C), heatwave (≥ 30·9˚C for 3 consecutive days).

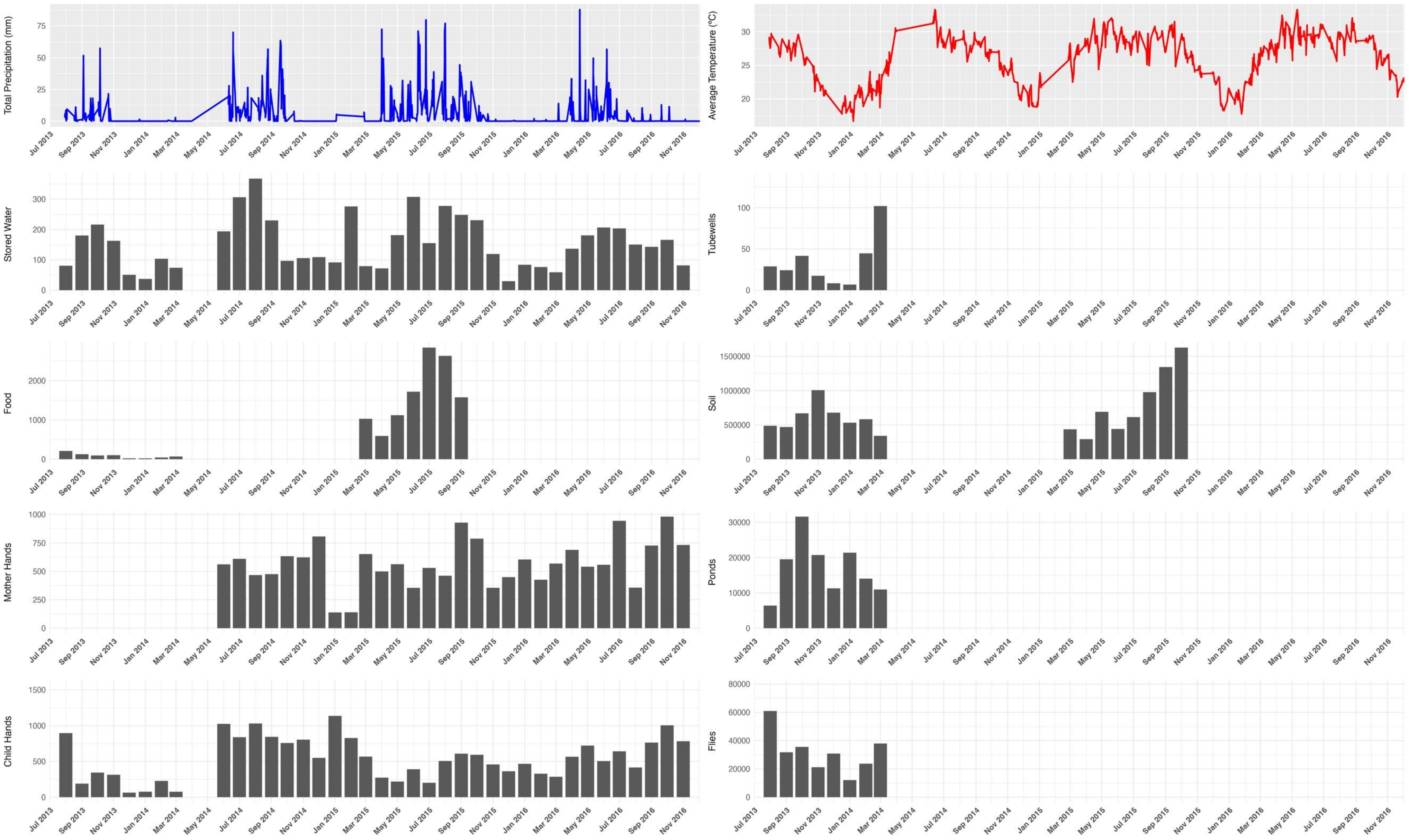

Figure S1. Daily precipitation and temperature, and monthly mean most probable number (MPN) of *E. coli* by sample type across the study period

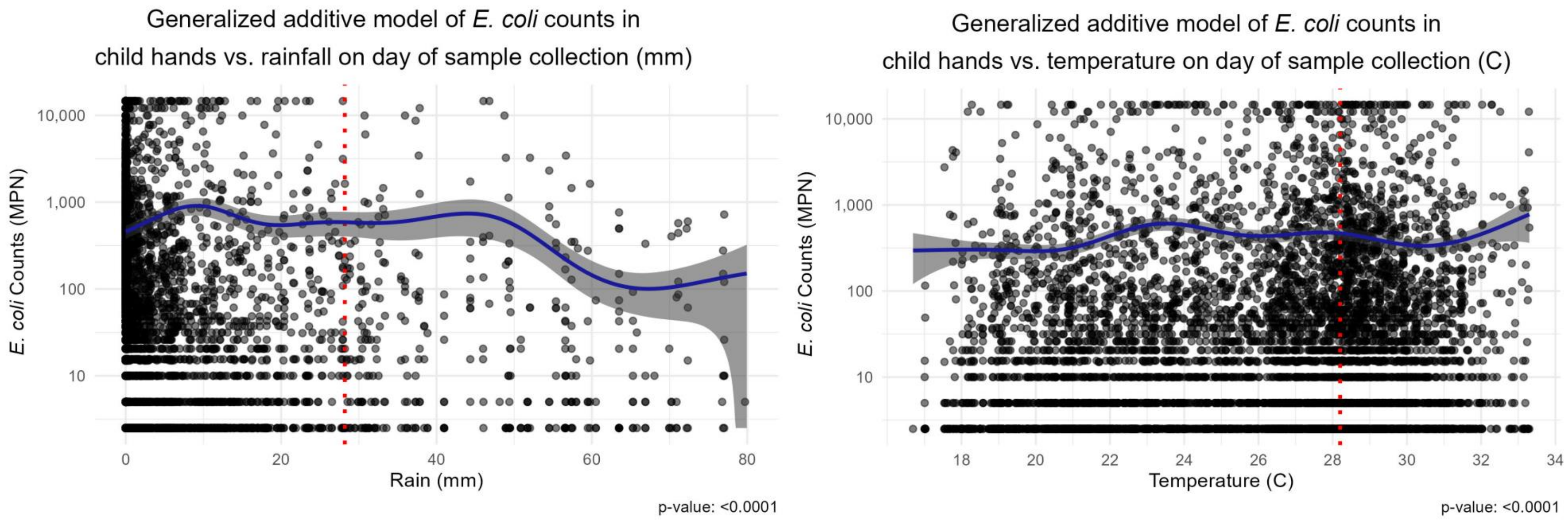

Figure S2. Adjusted generalized additive model plot of *E. coli* counts on child hands vs. continuous rainfall and temperature on the day of sample collection. The red dashed line corresponds to the 90th percentile (28·2 mm) for rainfall plots, and (30·2ºC) for temperature plots.

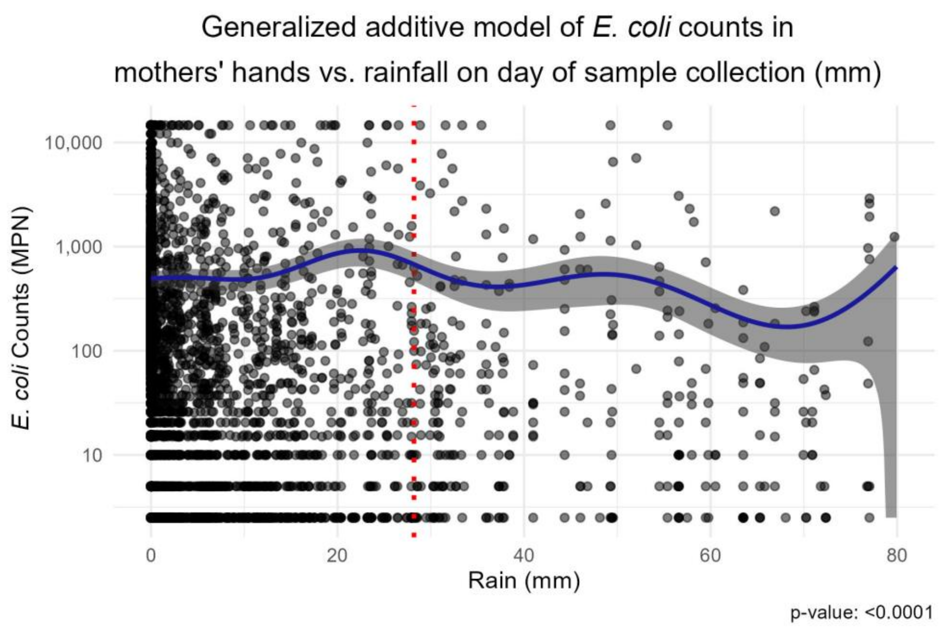

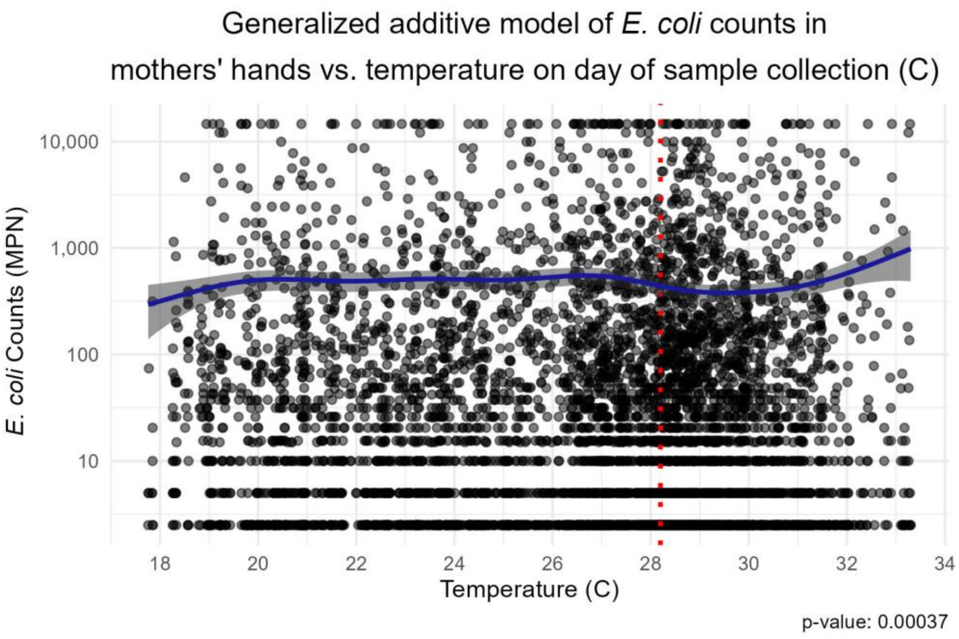

Figure S3. Adjusted generalized additive model plot of *E. coli* counts on mother hands vs. continuous rainfall and temperature on the day of sample collection. The red dashed line corresponds to the 90th percentile (28·2 mm) for rainfall plots, and (30·2ºC) for temperature plots.

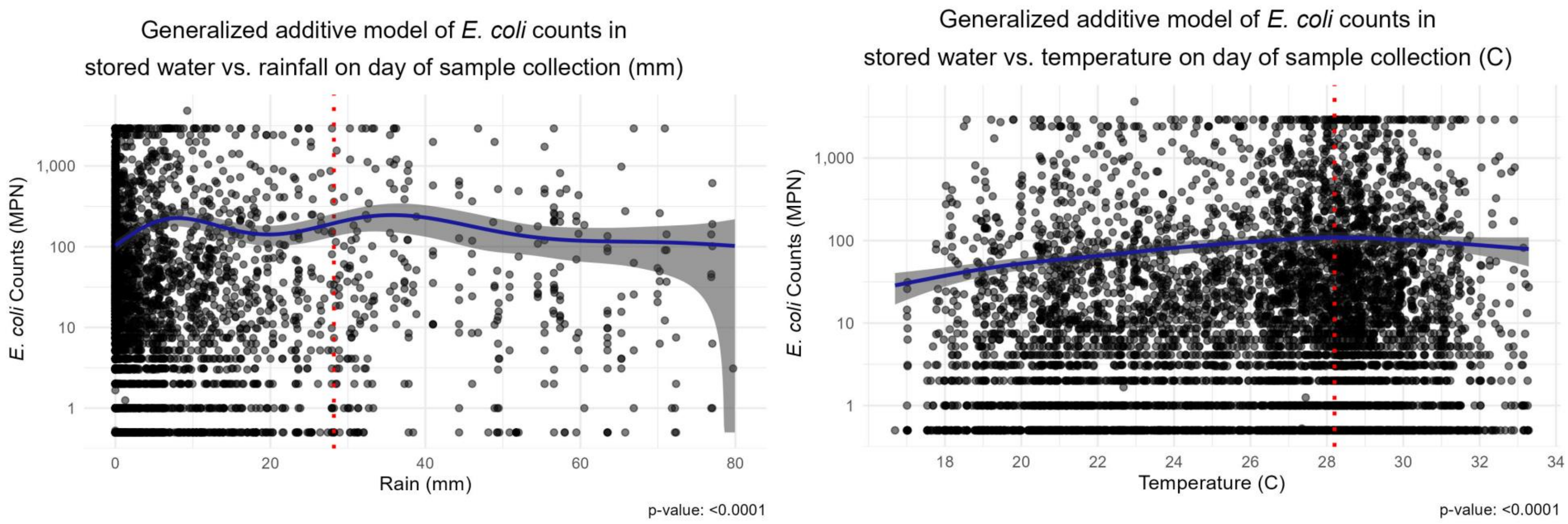

Figure S4. Adjusted generalized additive model plot of *E. coli* counts in stored water vs. continuous rainfall and temperature on the day of sample collection. The red dashed line corresponds to the 90th percentile (28·2 mm) for rainfall plots, and (30·2ºC) for temperature plots.

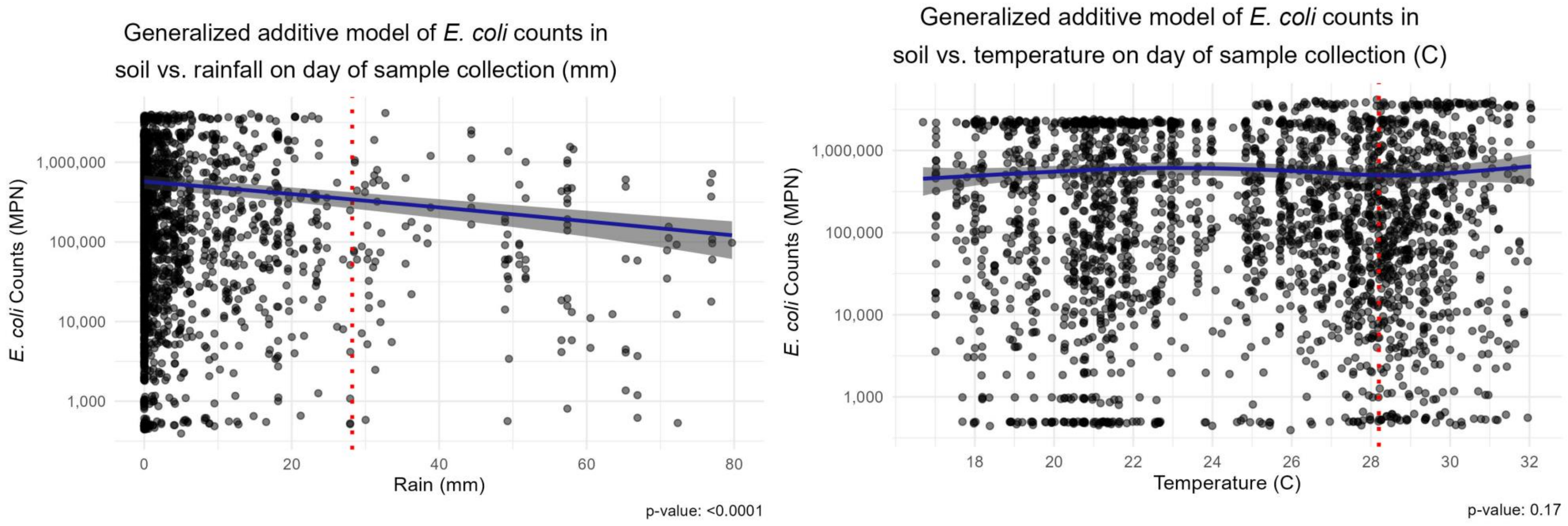

Figure S5. Adjusted generalized additive model plot of *E. coli* counts in soil vs. continuous rainfall and temperature on the day of sample collection. The red dashed line corresponds to the 90th percentile (28·2 mm) for rainfall plots, and (30·2ºC) for temperature plots.

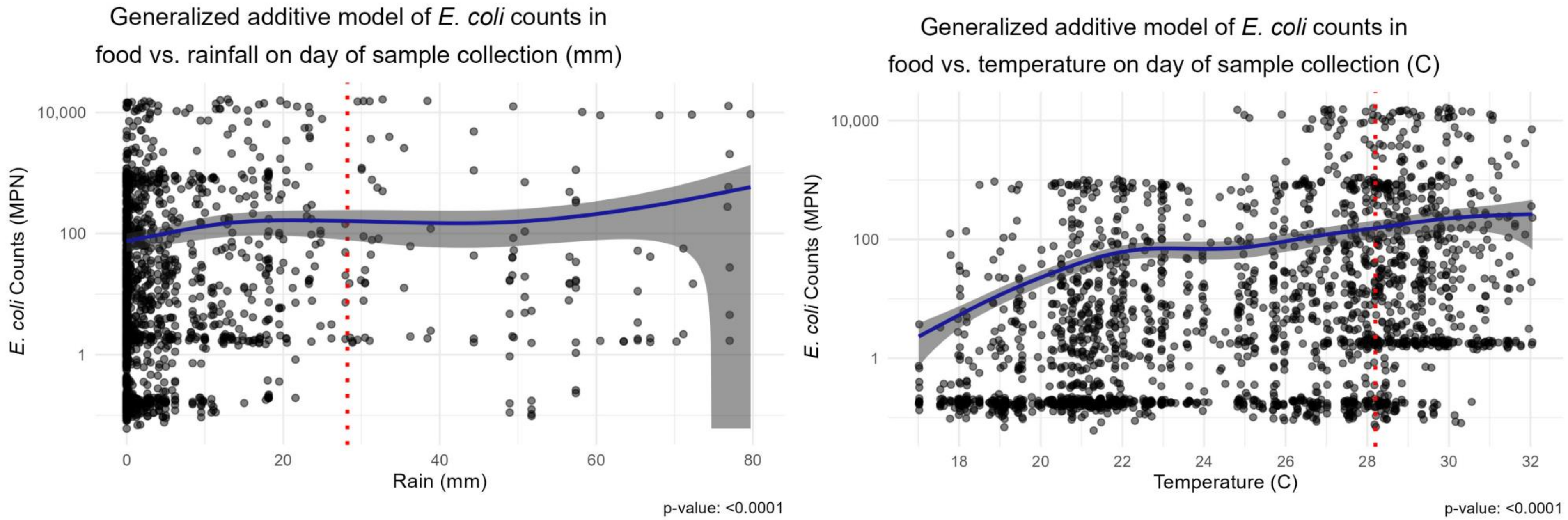

Figure S6. Adjusted generalized additive model plot of *E. coli* counts in food vs. continuous rainfall and temperature on the day of sample collection. The red dashed line corresponds to the 90th percentile (28·2 mm) for rainfall plots, and (30·2ºC) for temperature plots.

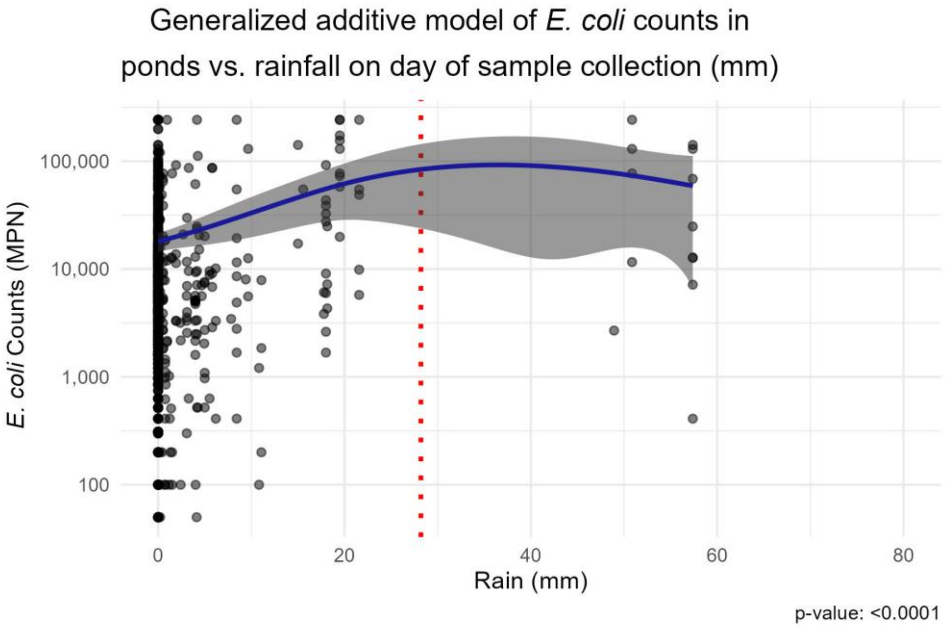

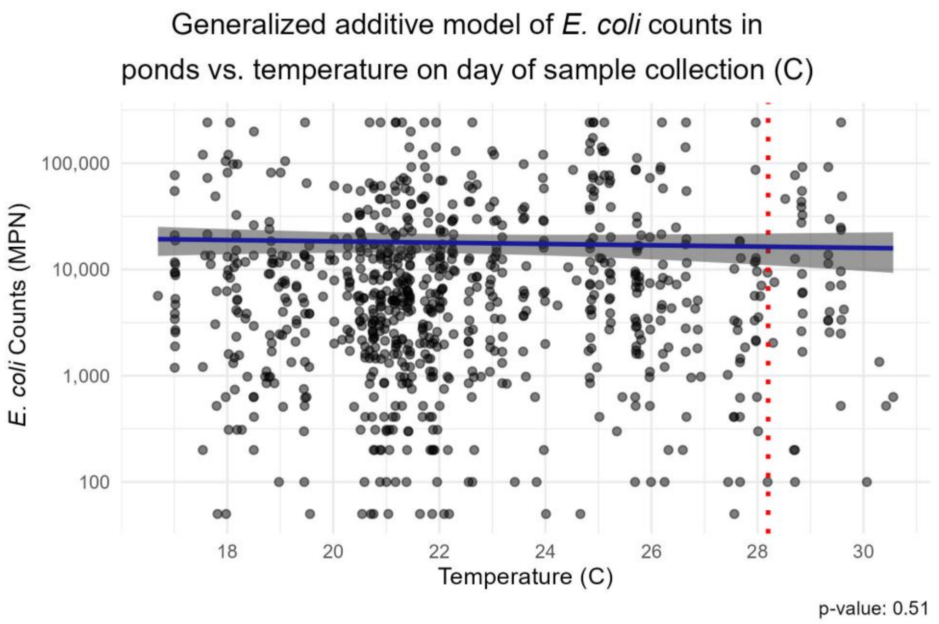

Figure S7. Adjusted generalized additive model plot of *E. coli* counts in ponds vs. continuous rainfall and temperature on the day of sample collection. The red dashed line corresponds to the 90th percentile (28·2 mm) for rainfall plots, and (30·2ºC) for temperature plots.

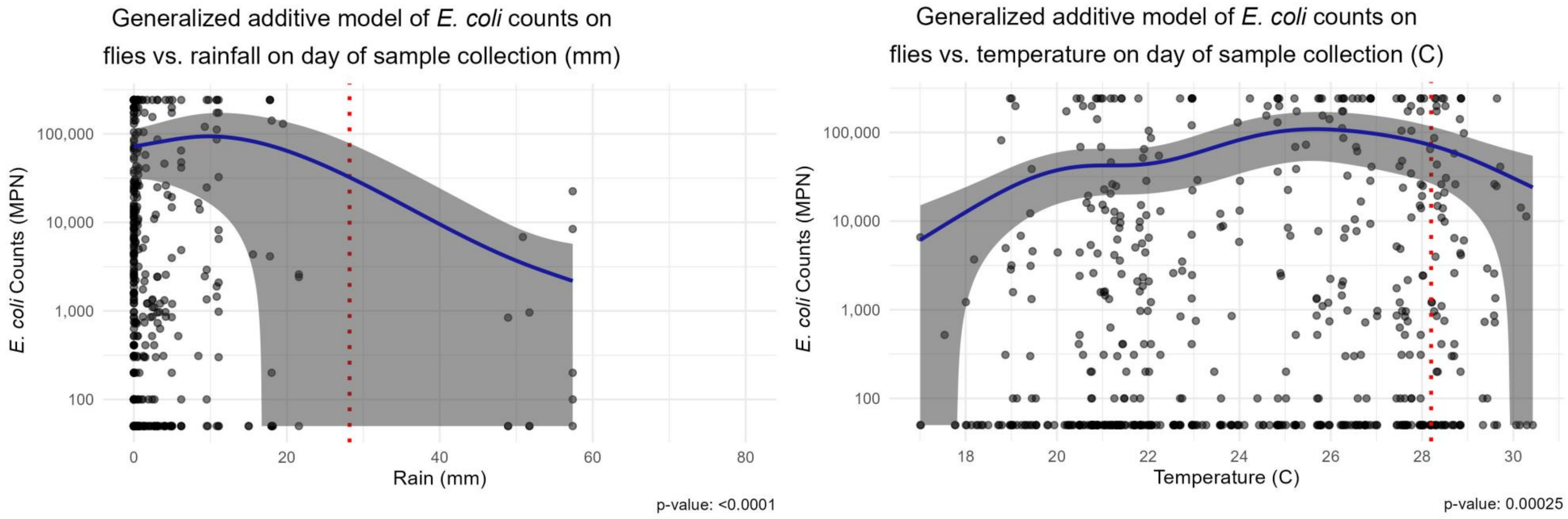

Figure S8. Adjusted generalized additive model plot of *E. coli* counts in/on flies vs. continuous rainfall and temperature on the day of sample collection. The red dashed line corresponds to the 90th percentile (28·2 mm) for rainfall plots, and (30·2ºC) for temperature plots.

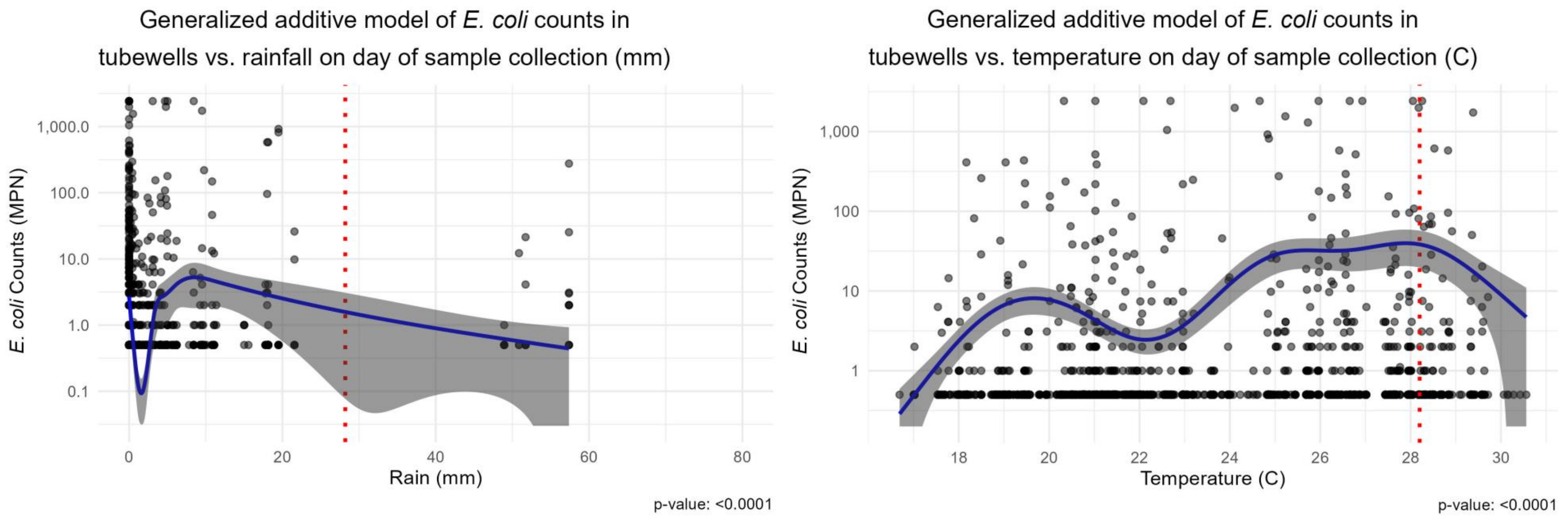

Figure S9. Adjusted generalized additive model plot of *E. coli* counts in tubewell water vs. continuous rainfall and temperature on the day of sample collection. The red dashed line corresponds to the 90th percentile (28·2 mm) for rainfall plots, and (30·2ºC) for temperature plots.

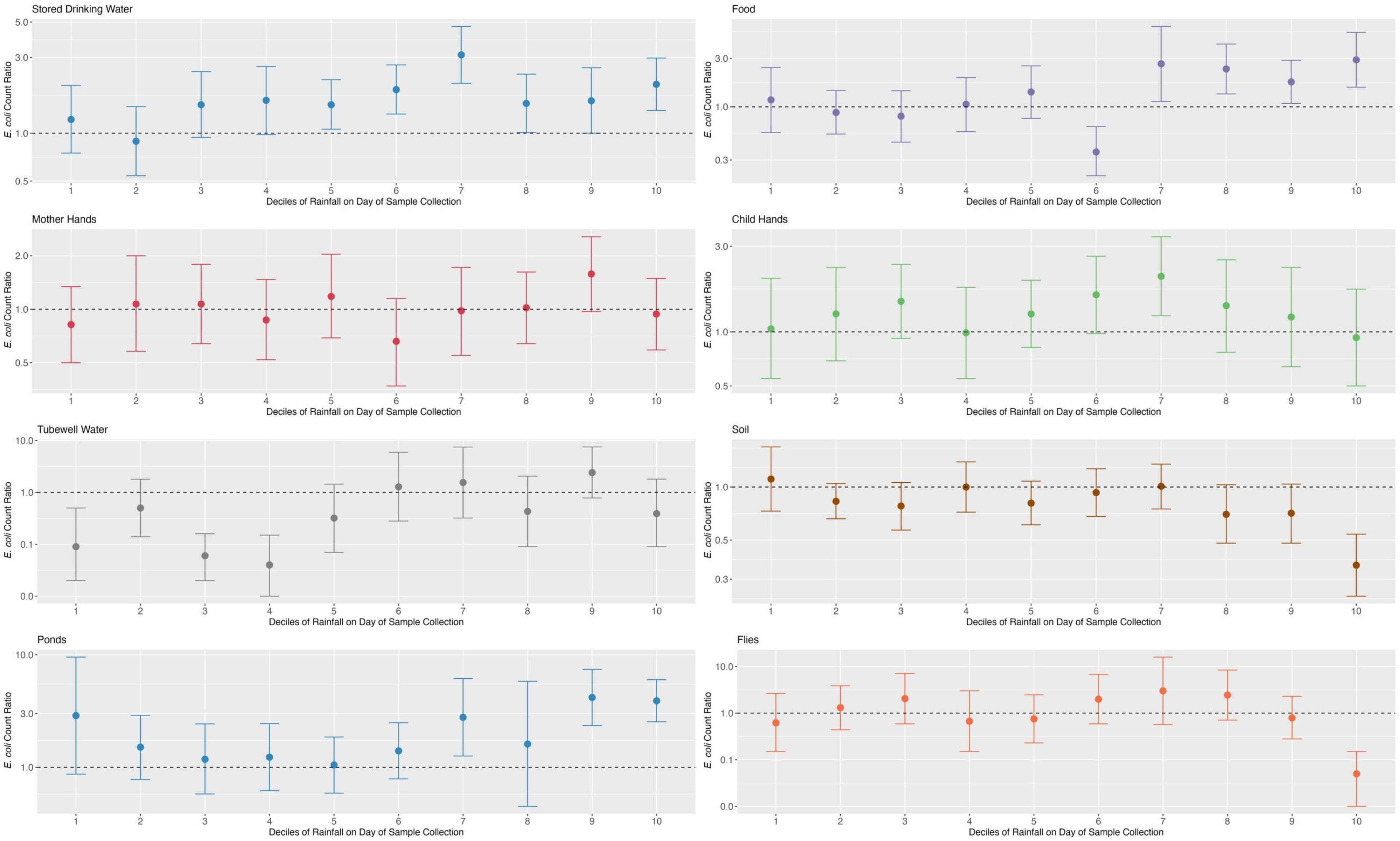

Figure S10. Adjusted *E. coli* count ratios associated with deciles of rain, compared to no rain, on the day of sampling

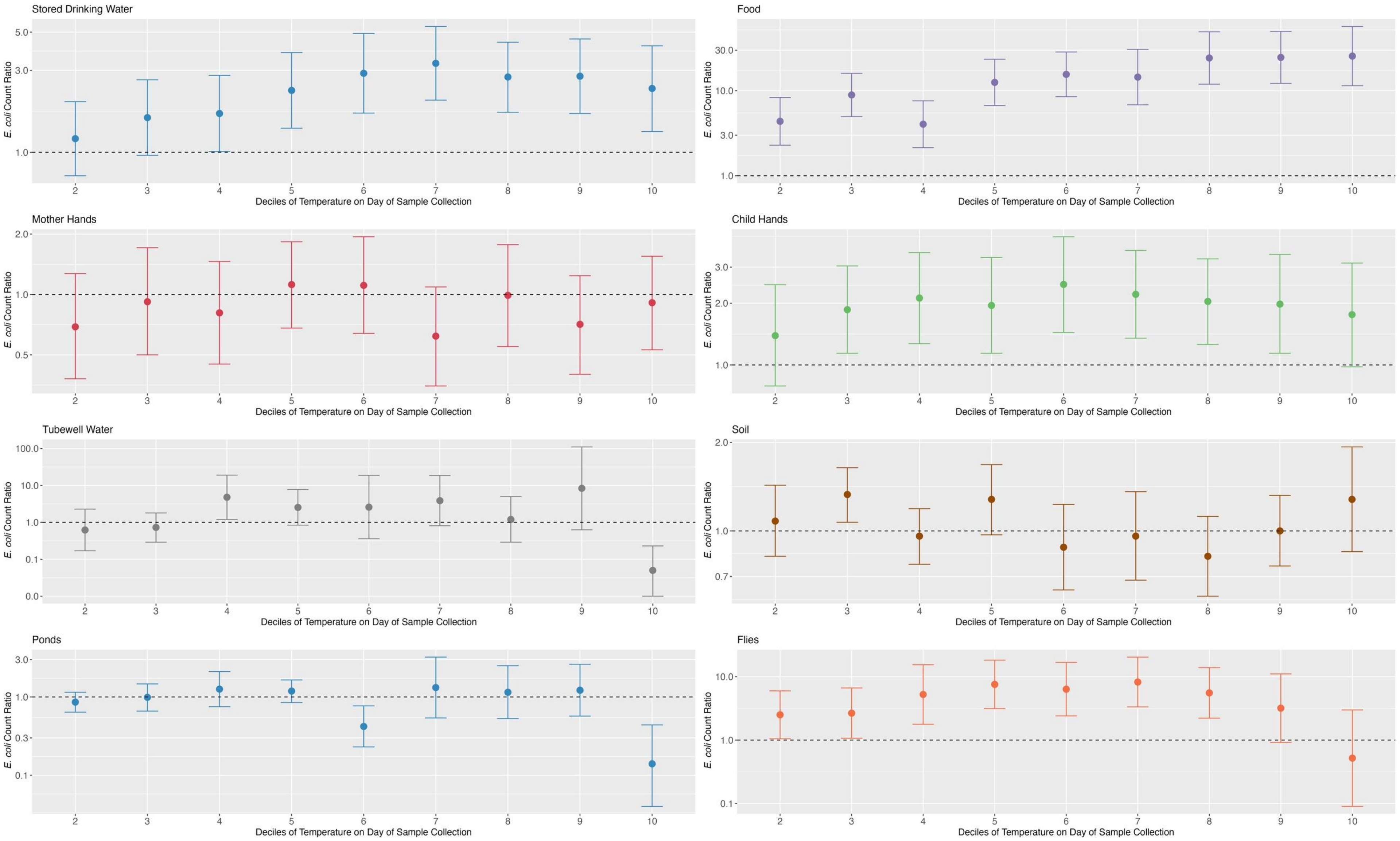

Figure S11. Adjusted *E. coli* count ratios associated with deciles of temperature, compared to 1st decile of temperature, on the day of sampling

Table S5. Adjusted[a] *E. coli* count ratios by sample type[b] associated with each weather category[c] across different antecedent timeframes

Adjusted	Stored Water (N=6350)		Food (N=2181)		Mother Hands (N = 5397)		Child Hands (N= 7092)		Source Water (N=1669)		Soil (N=2538)		Ponds (N=822)		Flies (N=610)	
	E. coli Ratio (95% CI)	p-value	*E. coli* Ratio (95% CI)	p-value	*E. coli* Ratio (95% CI)	p-value	*E. coli* Ratio (95% CI)	p-value	*E. coli* Ratio (95% CI)	p-value	*E. coli* Ratio (95% CI)	p-value	*E. coli* Ratio (95% CI)	p-value	*E. coli* Ratio (95% CI)	p-value
**Day of**																
No Rain	ref	--	ref	--	ref	--	ref	--	ref	--	ref	--	ref	--	ref	--
Some Rain	1.67 (1.29, 2.17)	< 0.0005	1.45 (0.89, 2.35)	0.357	0.96 (0.72, 1.28)	0.771	1.38 (1.09, 1.74)	0.008	0.36 (0.12, 1.07)	0.066	0.87 (0.73, 1.05)	0.140	1.52 (1.03, 2.25)	0.036	0.95 (0.27, 3.39)	0.941
Heavy Rain	1.674 (1.02, 2.64)	0.041	2.06 (1.22, 3.49)	0.007	1.62 (1.00, 2.60)	0.049	1.24 (0.68, 2.25)	0.478	1.93 (0.61, 6.13)	0.266	0.74 (0.52, 1.06)	0.101	4.46 (2.42, 8.20)	< 0.0005	0.94 (0.30, 2.99)	0.923
Extreme Rain	1.98 (1.36, 2.88)	< 0.0005	3.13 (1.63, 5.99)	0.001	0.90 (0.57, 1.41)	0.632	0.92 (0.52, 1.60)	0.757	0.34 (0.07, 1.76)	0.198	0.36 (0.24, 0.53)	< 0.0005	3.46 (2.34, 5.11)	< 0.0005	0.03 (0.01, 0.19)	< 0.0005
Below Median Temp	ref	--	ref	--	ref	--	ref	--	ref	--	ref	--	ref	--	ref	--
Above Median Temp	1.71 (1.39, 2.11)	< 0.0005	2.33 (1.60, 3.41)	< 0.0005	0.90 (0.70, 1.15)	0.38	1.22 (0.97, 1.54)	0.095	[c]	[c]	0.79 (0.67, 0.93)	0.004	[c]	[c]	[c]	[c]
Extreme Temp	1.49 (1.05, 2.12)	0.025	3.01 (1.51, 6.01)	< 0.0005	1.02 (0.72, 1.43)	0.927	1.02 (0.65, 1.62)	0.919	[c]	[c]	1.15 (0.82, 1.60)	0.412	[c]	[c]	[c]	[c]
**1 Day**																
No Rain	ref	--	ref	--	ref	--	ref	--	ref	--	ref	--	ref	--	ref	--
Some Rain	1.77 (1.37 , 2.29)	< 0.0005	1.19 (0.69, 2.07)	0.534	0.97 (0.73, 1.27)	0.798	1.03 (0.80, 1.34)	0.807	0.28 (0.12, 0.68)	0.005	0.95 (0.78, 1.17)	0.634	1.35 (0.93, 1.96)	0.111	0.91 (0.18, 4.75)	0.913
Heavy Rain	1.23 (0.81 , 1.87)	0.324	1.49 (0.80, 2.78)	0.475	1.44 (0.94, 2.20)	0.093	0.80 (0.50, 1.28)	0.345	0.58 (0.09, 3.51)	0.42	0.89 (0.68, 1.16)	0.367	2.91 (1.24, 6.82)	0.014	0.64 (0.15, 2.71)	0.549
Extreme Rain	2.25 (1.57 , 3.21)	< 0.0005	2.13 (1.19, 3.86)	0.011	1.18 (0.84, 1.66)	0.348	0.97 (0.63, 1.49)	0.877	0.10 (0.02, 0.62)	0.014	0.35 (0.25, 0.48)	< 0.0005	2.74 (1.51, 4.95)	0.001	0.28 (0.06, 1.39)	0.119
Below Median Temp	ref	--	ref	--	ref	--	ref	--	ref	--	ref	--	ref	--	ref	--
Above Median Temp	1.69 (1.35, 2.10)	< 0.0005	3.00 (1.99, 4.52)	< 0.0005	0.92 (0.70, 1.21)	0.554	1.25 (0.97, 1.61)	0.088	[c]	[c]	0.89 (0.75, 1.04)	0.145	[c]	[c]	[c]	[c]
Extreme Temp	1.62 (1.19, 2.22)	0.002	3.40 (1.71, 6.77)	< 0.0005	0.91 (0.66, 1.25)	0.565	0.93 (0.63, 1.37)	0.723	[c]	[c]	0.99 (0.73, 1.34)	0.959	[c]	[c]	[c]	[c]
**2 Days**																
No Rain	ref	--	ref	--	ref	--	ref	--	ref	--	ref	--	ref	--	ref	--
Some Rain	1.64 (1.27 , 2.10)	< 0.0005	1.06 (0.62, 1.83)	0.822	0.85 (0.65, 1.11)	0.233	1.00 (0.76, 1.32)	0.991	0.20 (0.08, 0.50)	0.001	0.93 (0.74, 1.16)	0.506	1.80 (1.32, 2.45)	< 0.0005	5.34 (2.40, 11.89)	< 0.0005
Heavy Rain	1.21 (0.82 , 1.81)	0.339	1.23 (0.68, 2.22)	0.49	1.24 (0.84, 1.83)	0.278	0.67 (0.43, 1.06)	0.087	0.21 (0.03, 1.62)	0.135	0.86 (0.66, 1.13)	0.280	3.36 (1.71, 6.57)	< 0.0005	3.99 (1.64, 9.68)	0.002
Extreme Rain	1.88 (1.34 , 2.65)	< 0.0005	1.40 (0.81, 2.42)	0.226	0.94 (0.67, 1.32)	0.717	0.88 (0.62, 1.27)	0.503	0.34 (0.01, 1.13)	0.079	0.46 (0.34, 0.61)	< 0.0005	2.36 (1.22, 4.57)	0.011	4.10 (1.16, 14.48)	0.028
Below Median Temp	ref	--	ref	--	ref	--	ref	--	ref	--	ref	--	ref	--	ref	--
Above Median Temp	1.58 (1.24, 2.01)	< 0.0005	3.12 (2.06, 4.72)	< 0.0005	0.99 (0.76, 1.30)	0.964	1.33 (1.01, 1.75)	0.042	[c]	[c]	0.88 (0.74, 1.06)	0.183	[c]	[c]	[c]	[c]
Extreme Temp	1.63 (1.20, 2.23)	0.002	4.40 (2.24, 8.65)	< 0.0005	0.91 (0.67, 1.24)	0.555	0.98 (0.68, 1.41)	0.900	[c]	[c]	0.94 (0.72, 1.24)	0.662	[c]	[c]	[c]	[c]
**7 Days**																
No Rain	ref	--	ref	--	ref	--	ref	--	ref	--	ref	--	ref	--	ref	--
Some Rain	1.42 (1.06 , 1.91)	0.019	0.59 (0.34, 1.02)	0.059	0.72 (0.53, 0.98)	0.039	0.88 (0.61, 1.28)	0.503	0.17 (0.07, 0.45)	< 0.0005	1.11 (0.83, 1.49)	0.494	1.14 (0.79, 66)	0.478	1.30 (0.51, 3.32)	0.582
Heavy Rain	1.47 (0.96, 2.23)	0.072	0.74 (0.36, 1.54)	0.426	0.96 (0.64, 1.44)	0.83	0.82 (0.53, 1.27)	0.372	0.07 (0.01, 0.47)	0.006	0.84 (0.61, 1.16)	0.283	2.34 (1.22, 4.49)	0.011	0.83 (0.26, 2.60)	0.744
Extreme Rain	1.77 (1.22 , 2.57)	0.002	0.81 (0.49, 1.34)	0.418	0.67 (0.48, 0.94)	0.02	1.13 (0.76, 1.68)	0.548	0.13 (0.02, 0.63)	0.012	0.66 (0.49, 0.89)	0.007	1.52 (0.88, 2.63)	0.135	2.62 (0.98, 7.04)	0.056
Below Median Temp	ref	--	ref	--	ref	--	ref	--	ref	--	ref	--	ref	--	ref	--
Above Median Temp	1.61 (1.25, 2.07)	< 0.0005	2.98 (1.87, 4.75)	< 0.0005	1.10 (0.84, 1.46)	0.482	1.27 (0.92, 1.74)	0.150	[c]	[c]	0.93 (0.77, 1.14)	0.497	[c]	[c]	[c]	[c]
Extreme Temp	1.74 (1.33, 2.28)	< 0.0005	4.39 (2.39, 8.05)	< 0.0005	1.00 (0.76, 1.32)	0.979	0.90 (0.63, 1.27)	0.533	[c]	[c]	0.74 (0.56, 0.97)	0.029	[c]	[c]	[c]	[c]
No Heatwave	ref	--	ref	--	ref	--	ref	--	ref	--	ref	--	ref	--	ref	--
Heatwave	1.15 (0.85, 1.55)	0.378	0.81 (0.47, 1.40)	0.452	0.81 (0.59, 1.11)	0.189	0.79 (0.50, 1.27)	0.334	[c]	[c]	0.54 (0.38, 0.78)	0.001	[c]	[c]	[c]	[c]
**14 Days**																
No Rain	ref	--	ref	--	ref	--	ref	--	ref	--	ref	--	ref	--	ref	--
Some Rain	0.85 (0.60, 1.21)	0.371	0.80 (0.53, 1.21)	0.295	0.78 (0.54, 1.15)	0.210	1.05 (0.71, 1.54)	0.809	0.26 (0.09, 0.75)	0.013	1.16 (0.92, 1.45)	0.204	1.15 (0.86, 1.53)	0.336	0.70 (0.36, 1.35)	0.287
Heavy Rain	0.81 (0.55 , 1.21)	0.312	0.64 (0.36, 1.14)	0.132	0.87 (0.51, 1.46)	0.591	1.18 (0.73 1.90)	0.504	0.11 (0.02, 0.50)	0.004	0.82 (0.61, 1.10)	0.182	1.99 (1.29, 3.07)	0.002	0.66 (0.28, 1.58)	0.352
Extreme Rain	1.04 (0.67 , 1.60)	0.869	0.65 (0.36, 1.15)	0.141	0.81 (0.49, 1.35)	0.422	1.27 (0.82, 1.97)	0.292	0.23 (0.05, 1.07)	0.062	0.78 (0.57, 1.05)	0.102	1.60 (0.99, 2.60)	0.057	1.91 (0.74, 4.90)	0.179
Below Median Temp	ref	--	ref	--	ref	--	ref	--	ref	--	ref	--	ref	--	ref	--
Above Median Temp	1.46 (1.12, 1.91)	0.005	1.96 (1.20, 3.21)	0.007	1.23 (0.90, 1.69)	0.189	1.07 (0.76, 1.50)	0.691	[c]	[c]	0.94 (0.74, 1.18)	0.584	[c]	[c]	[c]	[c]
Extreme Temp	1.52 (1.17, 1.96)	0.001	4.34 (2.56, 7.34)	< 0.0005	1.01 (0.73, 1.39)	0.944	0.78 (0.55, 1.11)	0.162	[c]	[c]	0.80 (0.63, 1.03)	0.088	[c]	[c]	[c]	[c]
No Heatwave	ref	--	ref	--	ref	--	ref	--	ref	--	ref	--	ref	--	ref	--
Heatwave	1.14 (0.86, 1.51)	0.359	1.58 (0.94, 2.66)	0.086	0.79 (0.57, 1.09)	0.149	0.74 (0.51, 1.08)	0.116	[c]	[c]	0.50 (0.36, 0.70)	< 0.0005	[c]	[c]	[c]	[c]

[a] Models for rainfall adjusted for temperature and vice versa. All adjusted models also controlled for the following variables: Binary intervention variable (intervention or control), sex of index child, age of index child (in days), number of children under the age of 18 in the household, number of people in the compound, mother's age (in years), mother's educational status, food security category (using HFIAS scale), minutes to water source, household having improved walls, household having improved floors, household wealth quintile based on owned assets, number of cows, goats and chickens/ducks in the compound, source water origin from a tubewell. Food models included hours since stored food was prepared and stored drinking water models included covered storage container, narrow-mouth storage container, and hours water has been stored.

[b] We could not estimate effects of extreme temperature and heat waves on source water (tubewells), ponds and flies due to data sparsity.

[c] Weather classifications are as follows: No rain (0 mm), some rain (<16·4 mm), heavy rain (≥16·4 and <28·2 mm), extreme rain (≥28·2 mm), below-median temperature (<27·1˚C), above-median temperature (≥27·1 and <30·2˚C), extreme temperature (≥ 30·2˚C), no heatwave (<30·9˚C), heatwave (≥ 30·9˚C for 3 consecutive days).

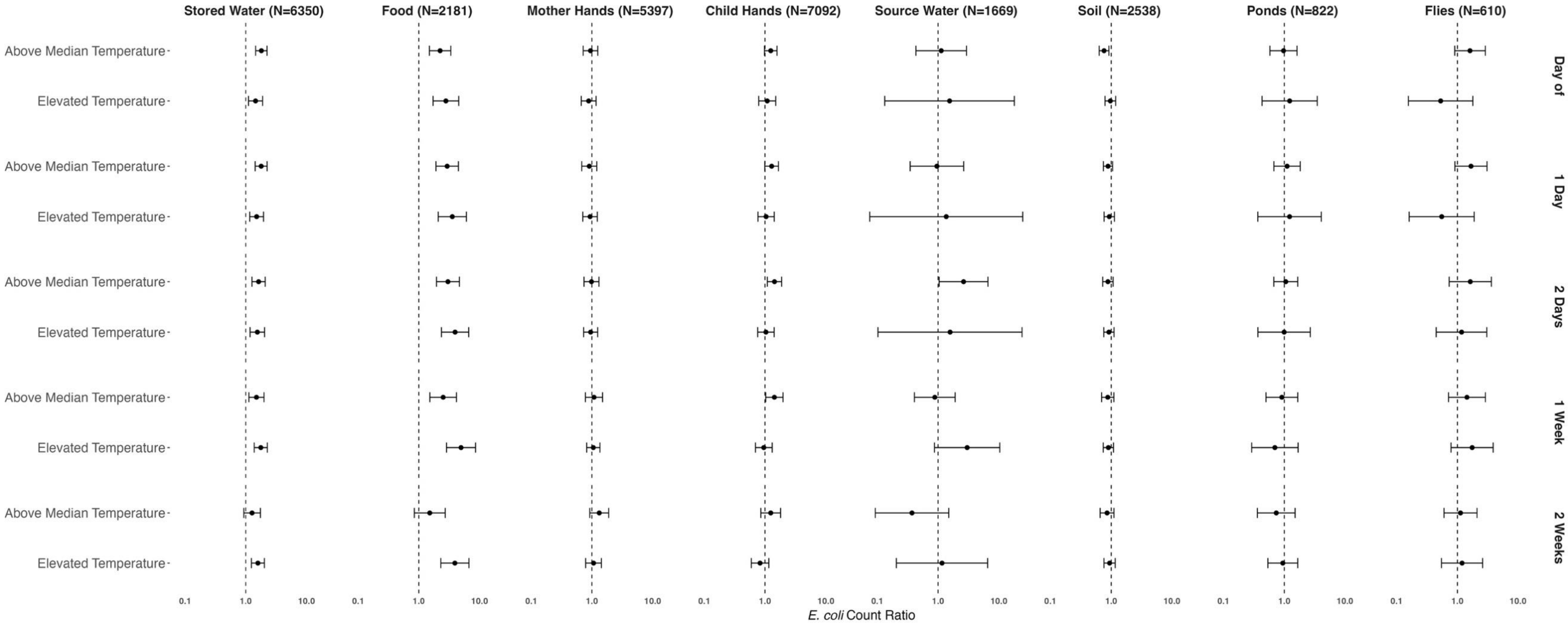

Figure S12. Adjusted *E. coli* count ratios by sample type associated with above-median and elevated temperature, compared to below-median temperature, during different antecedent periods. All adjusted temperature models controlled for the following variables: Rolling mean rainfall for the same antecedent period, binary intervention variable (intervention or control), sex of index child, age of index child (in days), number of children under the age of 18 in the household, number of people in the compound, mother's age (in years), mother's educational status, food security category (using HFIAS scale), minutes to water source, household having improved walls, household having improved floors, household wealth quintile based on owned assets, number of cows, goats and chickens/ducks in the compound, source water origin from a tubewell. Food models included hours since stored food was prepared and stored drinking water models included covered storage container, narrow-mouth storage container, and hours water has been stored. Weather classifications are as follows: below-median yemperature (<27·1˚C), above-median temperature (≥27·1 and <30·2˚C), elevated temperature (≥29·3˚C).

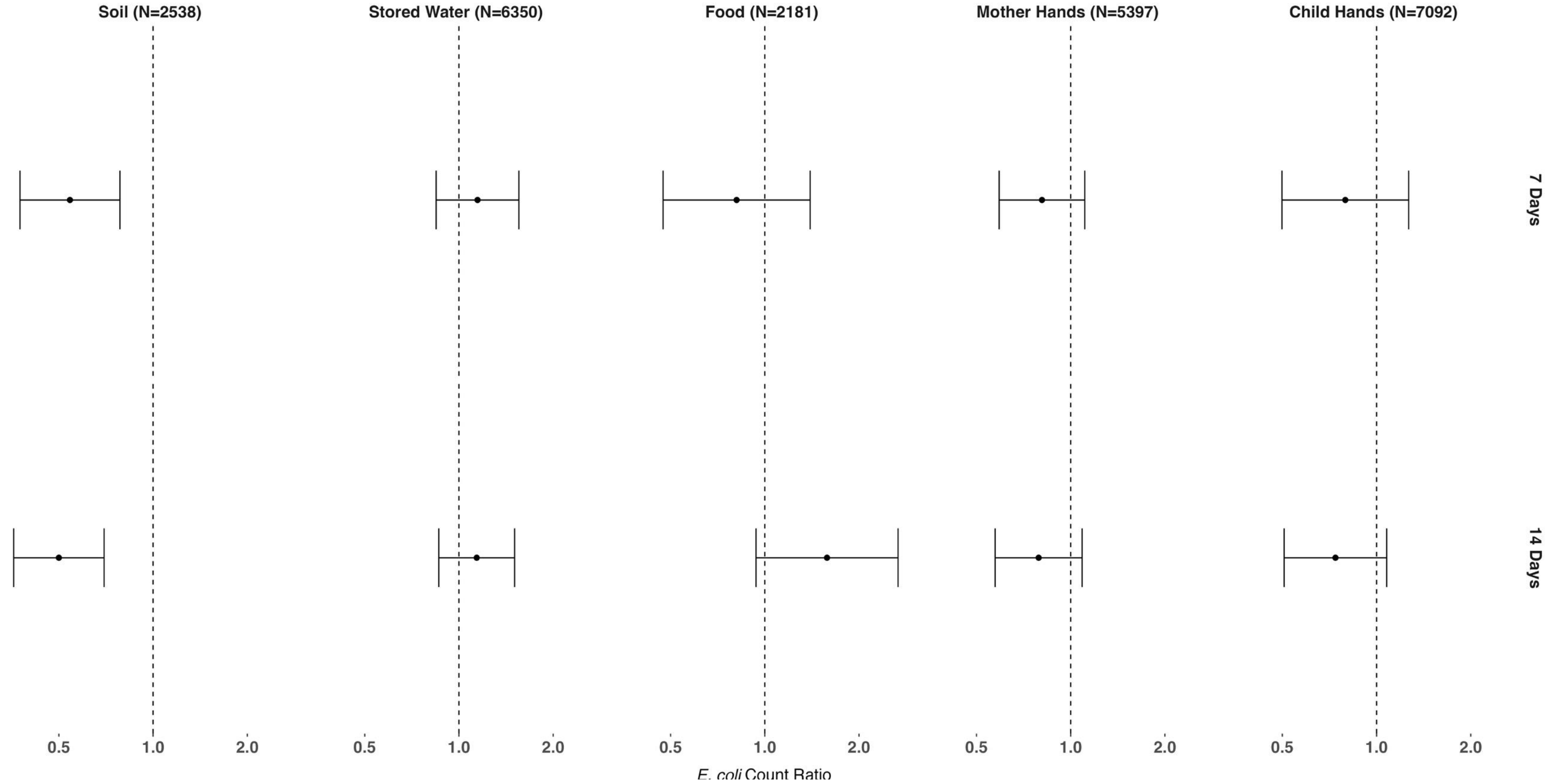

Figure S13. Adjusted *E. coli* count ratios by sample type associated with heatwaves within 7 and 14 days. All adjusted temperature models controlled for the following variables: Rolling mean rainfall for the same antecedent period, binary intervention variable (intervention or control), sex of index child, age of index child (in days), number of children under the age of 18 in the household, number of people in the compound, mother's age (in years), mother's educational status, food security category (using HFIAS scale), minutes to water source, household having improved walls, household having improved floors, household wealth quintile based on owned assets, number of cows, goats and chickens/ducks in the compound, source water origin from a tubewell. Food models included hours since stored food was prepared and stored drinking water models included covered storage container, narrow-mouth storage container, and hours water has been stored. Heatwave was defined as daily maximum temperature values >95th percentile (30·9°C) for three consecutive days. We could not estimate effects of heatwaves on source water (tubewells), ponds and flies due to data sparsity.

Table S6. Mean *E. coli* (MPN/100 mL) in stored drinking water by storage container type and rainfall, among samples stored for at least 8 hours

	Mean (SD)			
	Covered and narrow mouth	Uncovered and narrow mouth	Covered and wide mouth	Uncovered and wide mouth
Rainy days	100.4 (347.9)	410.6 (847.8)	199.2 (594.1)	618.6 (1088.3)
Dry days	64.9 (351.6)	251.3 (619.5)	89.9 (466.1)	141.3 (493.9)
